# TIME Impact – a new user-friendly tuberculosis (TB) model to inform TB policy decisions

**DOI:** 10.1186/s12916-016-0608-4

**Published:** 2016-03-24

**Authors:** R. M. G. J. Houben, M. Lalli, T. Sumner, M. Hamilton, D. Pedrazzoli, F. Bonsu, P. Hippner, Y. Pillay, M. Kimerling, S. Ahmedov, C. Pretorius, R. G. White

**Affiliations:** TB Modelling Group, TB Centre, London School of Hygiene and Tropical Medicine, Keppel Street, WC1E 7HT London, UK; Department of Infectious Disease Epidemiology, London School of Hygiene and Tropical Medicine, London, UK; Avenir Health, Glastonbury, CT USA; National Tuberculosis Control Programme, Ghana Health Service, Accra, Ghana; Aurum Institute, Johannesburg, South Africa; National Department of Health, Pretoria, South Africa; KNCV Tuberculosis Foundation, The Hague, The Netherlands; USAID, Washington, DC USA

**Keywords:** Capacity building, Mathematical modelling, Policy support, Tuberculosis

## Abstract

Tuberculosis (TB) is the leading cause of death from infectious disease worldwide, predominantly affecting low- and middle-income countries (LMICs), where resources are limited. As such, countries need to be able to choose the most efficient interventions for their respective setting. Mathematical models can be valuable tools to inform rational policy decisions and improve resource allocation, but are often unavailable or inaccessible for LMICs, particularly in TB. We developed TIME Impact, a user-friendly TB model that enables local capacity building and strengthens country-specific policy discussions to inform support funding applications at the (sub-)national level (e.g. Ministry of Finance) or to international donors (e.g. the Global Fund to Fight AIDS, Tuberculosis and Malaria).

TIME Impact is an epidemiological transmission model nested in TIME, a set of TB modelling tools available for free download within the widely-used Spectrum software. The TIME Impact model reflects key aspects of the natural history of TB, with additional structure for HIV/ART, drug resistance, treatment history and age. TIME Impact enables national TB programmes (NTPs) and other TB policymakers to better understand their own TB epidemic, plan their response, apply for funding and evaluate the implementation of the response.

The explicit aim of TIME Impact’s user-friendly interface is to enable training of local and international TB experts towards independent use. During application of TIME Impact, close involvement of the NTPs and other local partners also builds critical understanding of the modelling methods, assumptions and limitations inherent to modelling. This is essential to generate broad country-level ownership of the modelling data inputs and results. In turn, it stimulates discussions and a review of the current evidence and assumptions, strengthening the decision-making process in general.

TIME Impact has been effectively applied in a variety of settings. In South Africa, it informed the first South African HIV and TB Investment Cases and successfully leveraged additional resources from the National Treasury at a time of austerity. In Ghana, a long-term TIME model-centred interaction with the NTP provided new insights into the local epidemiology and guided resource allocation decisions to improve impact.

**Electronic supplementary material:**

The online version of this article (doi:10.1186/s12916-016-0608-4) contains supplementary material, which is available to authorized users.

## The need for a country-level modelling tool in TB

Tuberculosis (TB) is now the leading cause of death from infectious disease worldwide [1]. It is also a disease of the poor, with the majority of the burden carried by low- and middle-income countries (LMICs) and vulnerable populations [2, 3]. While overall incidence is falling, the current rate of decline will not enable countries to reach the targets set by the World Health Assembly [1, 4]. Despite this high burden, and the need for accelerated progress, national TB programmes (NTPs) in LMICs face substantial constraints on the resources available, and are therefore under high pressure to maximise the epidemiological impact (e.g. cases prevented, lives saved) with their limited means [5].

**Fig. 1:**
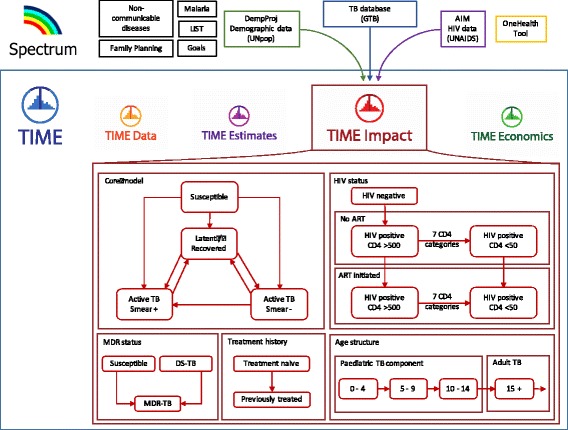
TIME Impact structure and link within Spectrum software suite. Figure illustrates how the TIME Impact tool is embedded in the Spectrum software suite, and linked to key modules and databases for demography, tuberculosis and HIV estimates (top row). The TIME box shows the basic model structure of TIME Impact (red boxes) and how TIME Impact fits within the other TIME modules

Modelling tools have been highly effective in supporting country programs to make more efficient policy choices as well as strengthening the case for investment to both domestic and international funders [6–8], for example, through applications to the Global Fund to Fight AIDS, Tuberculosis and Malaria (GFATM). Notable examples include the Spectrum software suite, which includes the AIM and Goals tools, which over the past decade have been extensively used to mobilise and direct resources in HIV (Fig. 1, top row) [9, 10], AEM which is used extensively in concentrated epidemics, and Optima, which specializes in allocative-efficiency.

Modelling capacity is limited in most LMICs, particularly in the area of TB. At the same time, policy decision-making is increasingly locally-led [11]. A pre-built, customisable TB modelling tool with a user-friendly interface could make modelling resources more widely available for policymakers in LMICs. By engaging in-country policymakers in the modelling process, local ownership of the modelling methods and results can be increased, as the process requires an assessment of the data and epidemic, including existing gaps, and working through the data and assumptions for potential interventions. Together, these benefits strengthen the rational foundation of TB policy decisions in LMICs [12]. In addition, a user friendly interface would allow for building local capacity, where local TB experts can progress from being informed consumers of modelling results to independent users of the modelling tool.

In TB, modelling is increasingly used to inform global [13, 14] and local TB policy [15, 16] on specific questions or areas [17–19]. However, TB models to date have been either limited in scope [17, 18] or developed for the use of academics and, as such, are difficult to access by NTPs as part of their planning process [14]. Therefore, there remains a need for a flexible user-friendly TB tool that could be customised to different epidemiological settings, and explore TB care and prevention activities across the NTP portfolio.

We developed TIME Impact as part of a flexible, free-for-download and user-friendly TB software package to provide an accessible and locally-owned modelling platform for in-country TB policymakers. In this paper, we describe the implementation and utility of the tool, as well as two case studies of its application in South Africa and Ghana.

### The TIME Impact tool

TIME Impact is implemented as part of the TIME modelling suite of software tools nested in the Spectrum software package (Fig. 1). As a component of Spectrum, TIME Impact automatically pulls in country data on TB (from the Global TB Programme (GTB) at the World Health Organization), HIV (from UNAIDS), and demography (based on estimates from the UN Population Division), which greatly facilitates customising the model to the national epidemiology (see Fig. 1, top 2 rows). Together with the other modules in TIME (TIME Data, TIME Estimates [20] and TIME Economics), TIME Impact enables NTPs and other TB policymakers, who may not have formal training in modelling, to better understand their own TB epidemic, plan their response, provide key inputs for funding applications and evaluate the implementation of the response.

### TIME Impact – epidemiological model

The core of TIME Impact is a dynamic compartmental transmission model which includes latent *Mycobacterium tuberculosis* infection and disease following recent (re)infection and reactivation (Fig. 1, top left red box) [14, 21]. To be useful, a principal requirement of the TIME Impact model is flexibility in order to allow calibration to different country settings, reflect historical local TB epidemiology, project likely future trends under current and alternative NTP intervention packages, as well as address critical policy questions. For this purpose, the model has been stratified by HIV and antiretroviral therapy (ART) status of individuals, their multi-drug resistance status, treatment history, as well as age to capture the different epidemiological characteristics of paediatric TB [22] (Fig. 1, lower right red box). Point value and ranges for natural history parameters are based on review of the literature (Additional file 1, section 6).

Critical for costing and understanding the value of diagnostic tools, TIME Impact also takes into consideration the population that is screened for TB. In order to approximate screening mechanisms, we apply a method similar to that developed by Menzies et al. [14]. The user-implemented screening algorithm thus results in true and false positive diagnoses, which after linking to care, gives rise to true and false positive notifications (see Additional file 1, section 4 for more details).

### TIME Impact – interface

**Fig. 2:**
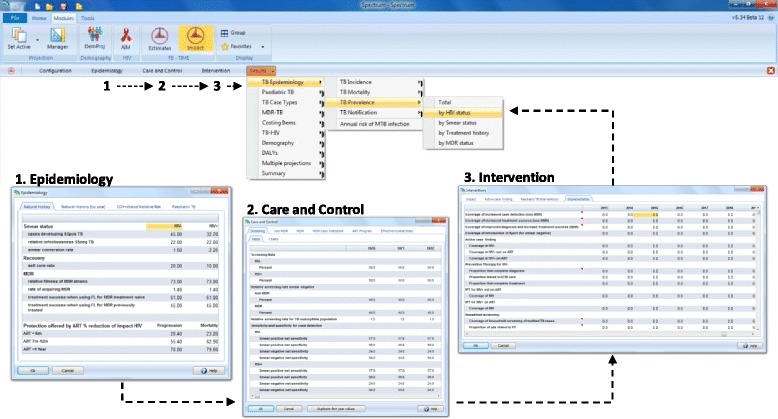
TIME Impact interface. TIME Impact’s user-friendly interface enables technical capacity building within National Tuberculosis Programmes. The user works through the different windows of (1) epidemiology, (2) care and control, and (3) interventions before visualising results (see drop down menus)

TIME Impact’s menu-driven interface improves the accessibility of the model and provides the opportunity to build technical capacity within NTPs, increasing the likelihood of local ownership of modelling results. Through the interface, users can explore the current epidemic as well as the epidemiological impact of NTP activities either by scaling-up specific TB care and prevention packages or exploring custom activities (Fig. 2).

The results window allows users to look at a variety charts and tables that contain model outputs over time, from changes in disease burden (e.g. prevalence, incidence, mortality), to specific TB epidemic dynamics (e.g. proportion latently infected, proportion due to recent transmission, annual risk of infection) and programmatic outputs (e.g. notifications, number screened, positive predictive value of the diagnostic algorithm). Through these outputs, users can see how the modelled epidemic changes over time, and whether historical trends are reflected sufficiently to increase confidence of the model’s projection of impact from future activities.

### Estimating the epidemiological impact of NTP activities

Activities to improve TB care and prevention can be modelled in two ways in TIME Impact, either by making use of the intervention window to incorporate potential NTP activities or by manipulating the care and control parameters to reflect the expected effect and scale-up of existing or alternative NTP activities.

Examples of pre-specified activities include periodic TB screening of people living with HIV (ART naïve, or on ART) followed by preventive therapy, or providing HIV testing and ART initiation for diagnosed TB cases; household contact screening, with the provision of isoniazid preventive therapy to under 5 year olds in contact with an index case.

Alternatively, users can capture the epidemiological impact of interventions by manipulating the care and control parameters in TIME Impact. Such activities include, but are not limited to, different clinic-based screening activities (i.e. expanding the population eligible for TB screening) and the roll-out of new diagnostic algorithms (which would be applied to those being screened).

It is important to emphasize that, for such custom interventions, a comprehensive dialogue between the modelling team and country stakeholders is critical to establish a shared understanding of the proposed activities, their expected effect and the data and assumptions that have been used to calculate this effect.

Further, to enable evaluation of a wide range of intervention activities, and to keep the tool as simple as possible, the level of detail within each intervention area is necessarily limited. Consequently, TIME Impact is not set up to address detailed operational questions on what diagnostic algorithm to use at each clinic level. Such questions could be tackled using other applications such as an ad-hoc operational tool, informed or guided by the WHO ScreenTB tool [18, 19]. TIME Impact can highlight the need for such an analysis, and incorporate the outcomes where relevant in the epidemiological model.

## Use of TIME Impact in-country

### Users and consumers of TIME Impact

TIME Impact has been designed as a modelling tool that can be used by non-modellers, e.g. trained TB epidemiologists such as selected NTP staff or (inter)national consultants. Users can then collaborate with key stakeholders and partners who know the local TB epidemiology, on how best to maximize access to the available data and effectively integrate within the policy decision process.

TIME Impact structure and results are tailored to ‘consumers’ within NTPs and other in-country policymakers, as they consider programming their TB-specific, or joint TB/HIV, response, as well as stakeholders and partners who support the process. These actors can make use of the TIME Impact results for the prioritisation of activities, preparation of a National Strategic Plan, and preparation of funding requests, either domestically to the Ministry of Finance or from bi-lateral and multi-lateral international donors.

Through training, which is enabled by TIME Impact’s user-friendly interface, key individuals can cross the line between informed consumers of TIME Impact modelling results towards independent users of the model, using it independently to address locally-generated questions, and take full ownership of and accountability for the results.

### TIME Impact as part of the NTP programming cycle

**Fig. 3:**
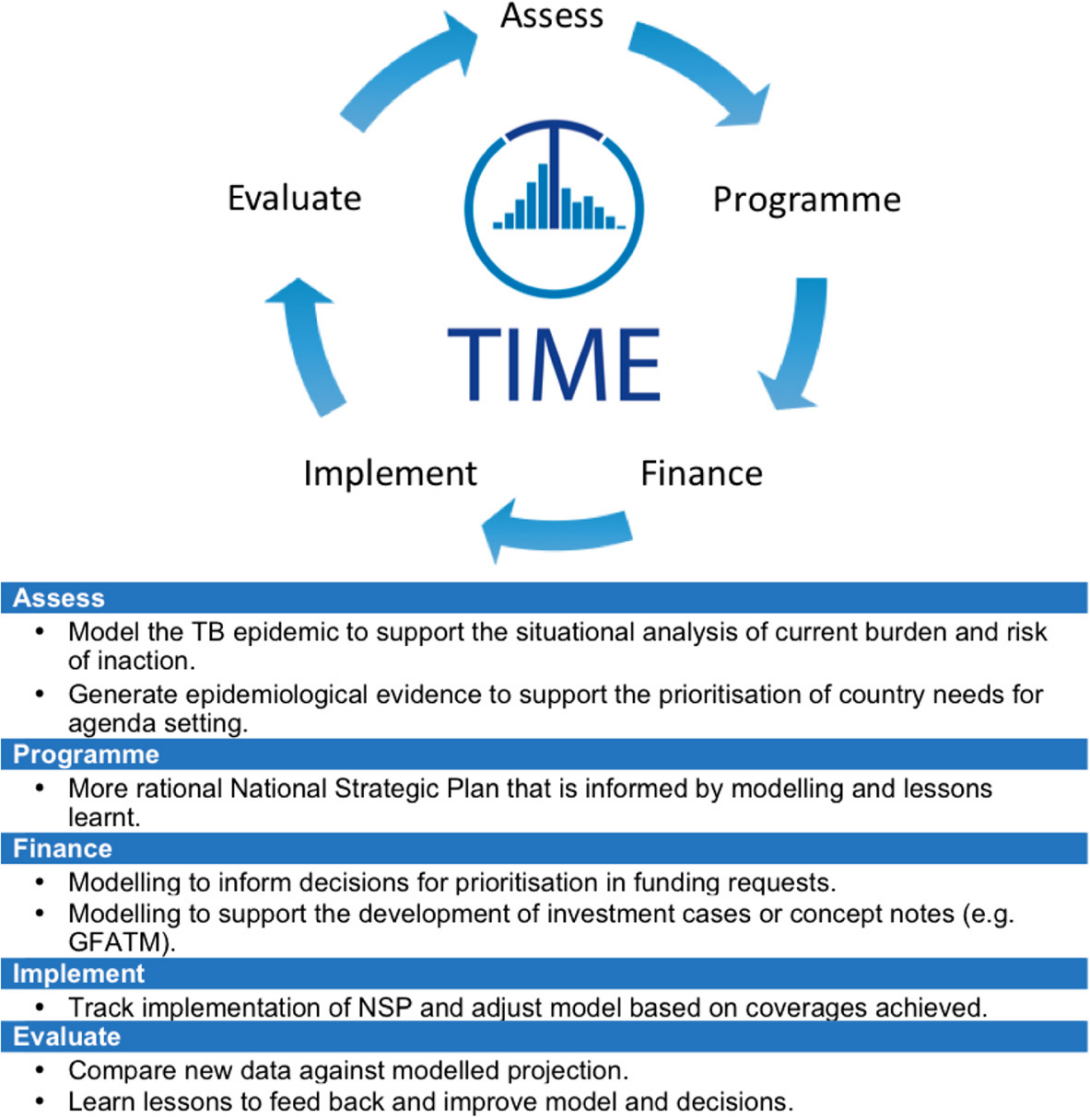
TIME Impact as part of the National Tuberculosis Programme (NTP) programming cycle. Figure and table illustrate how the TIME model can be a central focus of the NTP programming cycle and can support the process at each stage

In order to maximise the utility of TIME Impact, it should be used as part of the NTP programming cycle within a coherent decision-making framework, which links the model with relevant stakeholders and the country’s programming cycle of assessing the situation, planning a response, applying for funds, implementing interventions and evaluating their impact (Fig. 3) [23].

Modelling complements an epidemiological assessment of the impact of past activities and supports the situational analysis of current burden by generating epidemiological evidence to inform the prioritisation of country needs. During the planning phase, TIME Impact modelling can be used to explore the epidemiological impact of different potential activity programmes, which feed into resource allocation and allocative efficiency models. The process leads to a more rational NTP which has been informed by the modelling results and lessons learnt from, for example, the epidemiological assessment. When applying for funds, modelling can support the decisions for prioritisation of activities that are now considered fundamental to investment case analyses and funding applications such as those submitted as part of the GFATM concept note process. Modelling can be used to track the implementation of policies and coverages in the model can be adjusted based on coverages achieved. For evaluation, programmatic achievements, e.g. notifications achieved, can be checked against the modelled projection. Similar to the framework recently suggested by Knight et al. [12], a key feature is on-going engagement with all actors in the policy process, throughout the policy cycle. This cyclical health policymaking framework using modelling leads to a better understanding of the epidemic and a better understanding of the response for more effective policies that are supported by locally-generated evidence.

TIME Impact provides the opportunity to bring together different actors in the policy process who have a shared objective to reduce TB burden, but may offer diverse perspectives, allowing for a wider range of voices to be considered when interpreting evidence. These actors include governments (e.g. Ministry of Health), bilateral agencies (e.g. USAID, DFID), funding organisations (e.g. Global Fund) and academia (e.g. modelling experts), but this list is non-exhaustive [24].

The model serves as a focal point for discussions, with the TIME Impact model, current policies, and the evidence that support them at the centre of the dialogue for well-informed decision making. TIME modelling uses a mechanism that forces discussions on elucidating all assumptions, making the modelling and policy process transparent, thus building shared understanding of the results. Holding these discussions in a transparent way across the network of actors can be of great benefit to reach consensus, increase broad ownership of the modelling results, rationalise targets and objectives, and strengthen overall policy decisions [25].

### Data needs

When considering data requirements for TIME Impact, one needs to consider the natural history of TB, data for care and prevention (programmatic data, such as notifications, linkage to care, treatment success), and epidemiological data (e.g. prevalence or drug resistance survey) or estimates (e.g. incidence and mortality). Furthermore, data needs can be separated into inputs (data that get inputted into the model to influence the modelled epidemiology) and outputs (data that the modelled epidemiology are checked against). Table 1 highlights the data that are desirable, essential and automatically available.

**Table 1:** Data for TIME Impact

Included
• Demographic data and projections; UN Population Division
• Global Tuberculosis Programme (GTB) estimates for incidence, prevalence, mortality, notifications; GTB
• HIV burden and antiretroviral therapy (ART) coverage; UNAIDS
Required
• Estimated number of individuals screened (preferably trends); National Tuberculosis Programmes (NTPs)
• Diagnostic algorithms and coverage; NTP
• Linkage to care (trends, by multidrug resistance (MDR)); NTP, literature (MDR, GTB)
• Treatment success, by MDR (trends); GTB
• Drug susceptibility testing coverage; GTB
Desirable
• Prevalence survey results; NTP
• Drug resistance survey results; NTP
• HIV prevalence + ART coverage (required if high HIV burden setting); GTB, NTP
• Proportion of tuberculosis (TB) in children (<15 years old); NTP
• Current coverage and efficacy of TB programme activities; NTP
• Size of risk groups and TB prevalence; NTP

Table provides a non-exhaustive list of data used to inform TIME Impact and suggested sources. ‘Included’ data are automatically provided by Spectrum, whereas those listed under ‘required’ and ‘desirable’ need to be provided by the user

Through its links within Spectrum, TIME Impact automatically imports official national-level country data on TB (from GTB), HIV (from UNAIDS) and demography (from UN population division), to facilitate customising the model to the local setting. In some cases, sub-national level data are available, e.g. with UNAIDS HIV estimates, which facilitates the application of TIME Impact at sub-national level.

Care and control parameter data are also model inputs, which come from programmatic data that describe the screening algorithms used, what proportion of patients diagnosed with TB are linked onto care and successful treatment outcomes.

Where possible, context specific data on the expected epidemiological impact of interventions is also desirable, though often unavailable for TB.

### Country case studies of TIME Impact

The TIME Impact software tool is now available for free download at http://www.TIMEmodelling.com. It has been applied successfully at various points in NTP programming cycle, exploring different aspects of the tool’s functionality. Across divergent settings in terms of epidemiology and policy debate, TIME Impact was able to capture the local TB epidemic and reflect historical trends, and projections were used to guide policy discussions. Table 2 demonstrates how the model is able to match target data when calibrating to the TB epidemic in two divergent epidemiological settings, using South Africa and Ghana as examples.

**Table 2:** Model fit to calibration targets

	South Africa		Ghana	
	Target (2012)	Model	Target (2013)	Model
Notifications rate (per 100,000)	667	622.7	61.9	60.3
Prevalence rate (per 100,000)	705 (388–1114)	662	290 (113–548)	312
Incidence rate (per 100,000)	900 (832–990)	892	168 (81–286)	167
Mortality rate (per 100,000)	179 (149–212)	191	52 (24.8–88)	64.6
% prevalence MDR (treatment naïve)	1.8 (1.5–2.3)^a^	1.7	1.9 (0.1–5.3)	2.9
% prevalence MDR (retreatment)	6.7 (5.5–8.1)^a^	6.1	20 (0.1–40)	13.7
15+ HIV prevalence	15 (14–16)	15.4	1.5 (1.2–2.0)	1.34
ART coverage	36 (34–39)	34	32 (24–41)	27.5

^a^ South Africa MDR prevalence based on 2002 survey data

ART, Antiretroviral therapy; MDR, Multidrug resistance

### South Africa: link to policy and capacity building

**Fig. 4:**
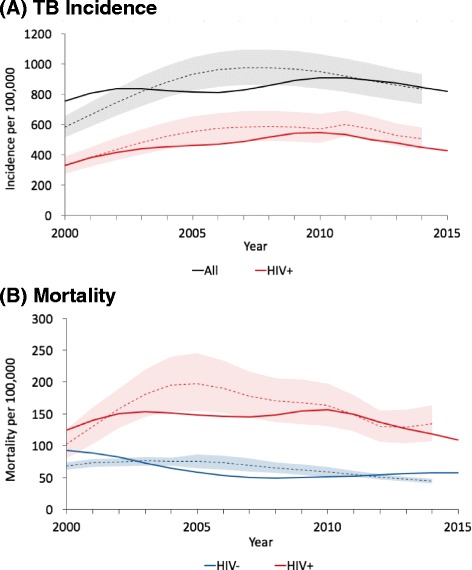
Model outputs for tuberculosis (TB) incidence and mortality in South Africa. The calibration focussed on matching 2012 data and aimed to fit within the confidence intervals around the Global TB Programme (GTB) estimates (thin solid lines). **a** TB Incidence: Modelled incidence (thick solid line) closely matches GTB estimates (dotted line). Model matches disaggregation by HIV status and annual decline in incidence in 2012. **b** Mortality: Modelled mortality (thick solid lines) match GTB estimates in 2012 (dotted line)

In South Africa, TIME Impact has been applied to provide evidence for the TB component of the country’s first-ever TB investment case, where the modelling results are instrumental in informing governmental TB spending from 2016 onwards. Figure 4 shows modelled outputs for baseline incidence and mortality of the TB epidemic in South Africa. The software tool has also been used as part of a South African capacity building project at the national and provincial level, which aims to integrate the use of a modelling framework into sub-national TB policy discussions. This local ownership and direct link to policy stands in contrast to other models that have investigated TB epidemiology and interventions in the South African context. While there is a substantial amount of modelling activity occurring in South Africa [13, 14, 26], none of these are run directly by locally-trained National or provincial TB Programme members. This case study shows that, through local ownership, we can better influence decision making, for example through additional funding for TB in 2017–2019 as part of a combined TB/HIV conditional grant.

### Ghana: reprogramming the TB response

In 2013, Ghana undertook a national TB prevalence survey which showed a generalised epidemic that is four times higher than previously estimated (all forms prevalence 290 vs. 71 per 100,000). The initial focus of the operational plan was to shift from passive screening towards active case finding in high-risk groups.

The TIME modelling framework was applied in-country to support decision-making and setting priorities within the NTP and the Global Fund Country Team. This work is part of an on-going collaboration with in-country policymakers and international stakeholders as the NTP goes through the process of reprogramming their national response.

**Fig. 5:**
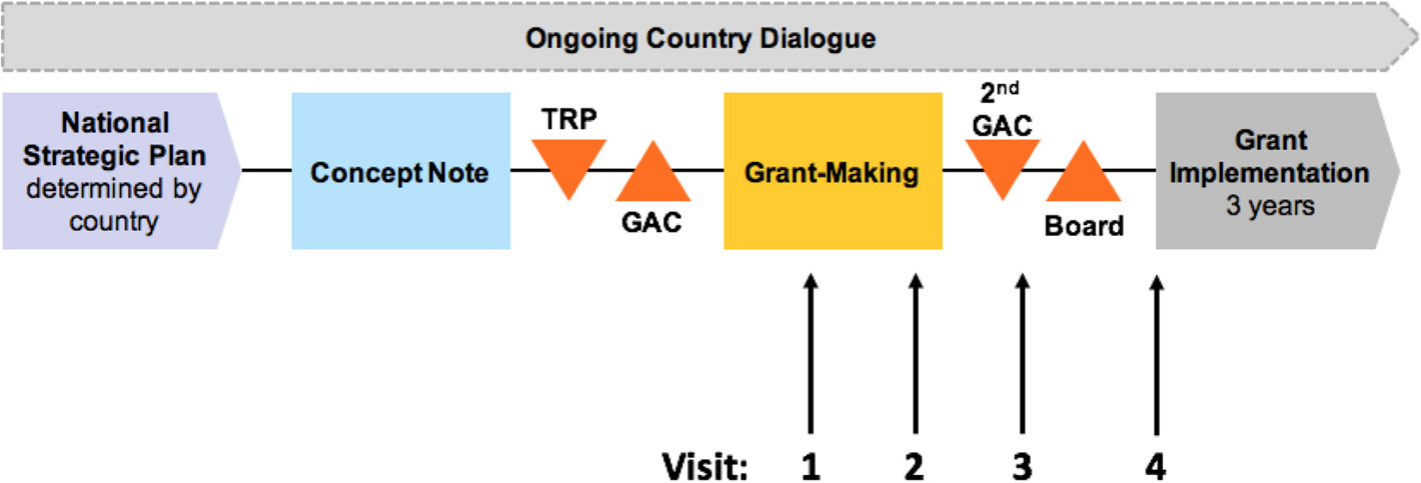
Global Fund to Fight AIDS, Tuberculosis and Malaria New Funding Model and country engagement timeline. TRP, Technical review panel; GAC, Grant approval committee. Figure adapted from the Global Fund to show visits to Ghana along the New Funding Model

Discussions around the TIME Impact modelling results informed during the grant-making phase of the Global Fund’s New Funding Model. The discussions were continued through repeated visits to establish a long-term collaboration with the NTP and provide continued support along the New Funding Model process (Fig. 5).

**Fig. 6:**
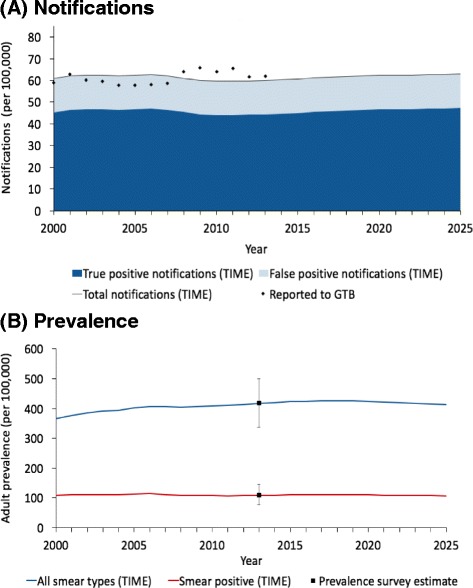
Model outputs for notifications and prevalence in Ghana. **a** Notifications: Total notifications from model (thin solid line) closely match Global Tuberculosis Programme data (black dots). TIME Impact estimates the positive predictive value amongst notifications to be 75 %. True positive notifications are shown in the dark blue shaded area and false positive notifications are shown in shaded light blue. **b** Prevalence: Model was calibrated to adult prevalence estimates from the 2013 national prevalence survey (squares). Modelled smear positive adult prevalence is represented in red and all forms adult prevalence is represented in blue

The TIME modelling framework was used to provide a clear understanding of the current and future epidemic, given the new prevalence survey results and in the absence of further action (Fig. 6). This showed that, in the absence of additional NTP activities, the prevalence of TB remains stable and may even increase in future years.

The TIME Impact modelling results highlighted a high risk of not reaching the ambitious national notification targets stated in the country’s performance framework as part of the Global Fund grant. The application of the TIME modelling framework in-country contributed to the decisions to shift focus of available resources from active screening in high-risk groups towards improving clinic-based screening and an expansion in coverage from 42 % to 100 % of districts.

The TIME modelling framework is continuing to be applied as the Ghana NTP moves towards implementation of their reprogrammed response.

### Other country-support experiences

In addition to these examples, TIME Impact has been applied in various other country collaborations, in particular for the purpose of strengthening the case for investment in country’s Concept Notes for GFATM applications, e.g. for Sudan, Bangladesh and Viet Nam. In Viet Nam, TIME Impact contributed to the narrative of the Concept Note to the GFATM, which was highly successful.

### Impact evaluation of implementing TIME

Identifying the specific programmatic effect of a policy change is both critical as well as challenging, further compounded by the complexity of attributing part of that effect to a specific input to the complicated policy process. Efforts are currently underway to quantify the impact from implementing TIME in Ghana and South Africa, results of which are expected in the coming years.

### Future development

While the country-examples show that TIME Impact has been useful in its current form, development is ongoing to expand the model functionalities in line with feedback from users and policymakers, and address current limitations.

One key limitation of the current version of the TIME Impact tool is the assumption of homogenous mixing. While common in epidemiological models of TB, there are notable exceptions around age-specific mixing [27] and poverty [28]. Development is ongoing to introduce the facility in TIME Impact, which would process information on the relative burden of TB in the general and at-risk population and the mechanism behind this higher burden and, critically, the level of contact between groups [28]. Further, as extensive drug resistance becomes an increasingly large problem, we will look to extend our drug-resistant strata to include the development of extensively drug-resistant TB.

Finally, all epidemiological modelling results are uncertain and it is important to convey this uncertainty to policymakers [6]. Development is ongoing for an automated framework to facilitate fitting the model to epidemiological data and generating uncertainty bounds around results introduced by assumptions regarding natural history, epidemiological data, the epidemiological effect of interventions and what happens in the future.

Another key area of interest is capturing the epidemiological impact of socioeconomic trends and structural determinants in the population. The End TB Strategy places increased emphasis on these issues [29], but gaps in both data and technical understanding currently prohibit confident modelling and this is not currently possible in TIME Impact. Future model and knowledge development should allow us to incorporate such functionality in due time.

### Costing and resource allocation

Strategic planning requires relating the cost of TB interventions to their epidemiological impact. National TB Programmes, Ministries of Health and Finance, NGOs and international donors must be able to formulate and answer a variety of questions about the relative impact of different intervention scenarios in order to maximize allocative efficiency, estimate cost effectiveness metrics such as cost per disability-adjusted life year or death averted, and accurately estimate the budget requirements and funding gaps associated with meeting strategic targets or implementing new programmes.

TIME Impact and TIME Estimates are linked to the OneHealth Tool, a comprehensive costing and budgeting tool developed by a group of UN agencies, including WHO, UNAIDS, UNDP, UNFPA, UNICEF and the World Bank. OneHealth provides a single framework for planning, costing, impact analysis, budgeting and financing of strategies for major diseases and health system components. OneHealth’s TB costing module is designed to mimic the WHO TB Planning and Budgeting Tool, a detailed ingredients-based costing tool developed by the Global TB Programme [30]. Users can control the coverages of diagnostic, treatment and patient support interventions over time, modify the population targeted to receive each intervention, cost the construction of new laboratories, and match budget lines to fit with national or international funder requirements.

Development of a new TIME Economics module is currently underway. TIME Economics is intended to address TB-specific allocative efficiency and cost-effectiveness questions.

## Conclusion

In summary, the TIME Impact software tool is now available and has advanced the field of modelling to support TB policy discussions at country level. As development continues in collaboration with stakeholders from the TB community, the focus remains to integrate capacity building with generating modelling results that have a high local ownership, now considered for policy discussions at the national and sub-national levels.

## Additional file

Additional file 1: TIME Impact Technical Appendix. (PDF 783 kb)
