# Supplementary material for: TIME Impact – a new user-friendly tuberculosis (TB) model to inform TB policy decisions
Source: BMC Med. 2016 Mar 24;14:56. doi: 10.1186/s12916-016-0608-4 (PMC4806495; doi:10.1186/s12916-016-0608-4)
Supplement: Additional file 1: — TIME Impact Technical Appendix. (PDF 783 kb) [file 12916_2016_608_MOESM1_ESM.pdf]

TIME Impact: Technical Appendix

Table of Contents

1. Overview 2
2. TB Model Core 2
3. Age structure 5
4. Model strata 5
Treatment History strata 5
MDR strata 6
Interactions between treatment history and MDR strata 8
Integration of HIV model 11
Screening for disease 13
5. Full model equations (excluding HIV strata and interventions) 14
Table 1. Legend for model parameters used in equations 14
Model equations 15
6. Model parameters 18
Default value and ranges for TIME Impact model parameters 18
Table 2. Default values for parameters 18
7. Interventions 24
Methods 24
Intervention Matrix 24
Increased Case Detection (non-MDR) 24
Increased Treatment success (non-MDR) 24
MDR – increased diagnosis, linkage and treatment success 24
IPT for HIV positive individuals 24
Increased ART coverage 25
HIV testing and ART initiation 25
Active Case Finding 25
Preventive Therapy for HIV negative individuals 26
IPT for child household contacts of under 5 years old and ACF among household members of all ages 26
8. Summary health measures 28
9. Demography 29
10. Model Initialisation and population size adjustments 30
Model initialisation 30
11. Fitting 30
12. References 31

1. Overview

This is a template for a deterministic compartmental model of TB transmission which is similar in structure to a number of previously published models. The model assumes that different strata mix homogenously (i.e. random mixing). The core TB model structure is shown in Figure 1. Model parameters (see Table 1 and 2) are based on previously published estimates, and are reviewed periodically. Interventions are also described.

2. TB Model Core

Disease progression

Susceptible (S) individuals are infected at a rate $\lambda = \beta(I+c*N) / T$ where β is the effective contact rate, I the number of smear positive and N the number of smear negative TB cases. c is the relative (lower) infectiousness of smear negative cases compared to smear positive cases, and T is the total population. A proportion α of newly infected individuals develop primary TB, a proportion (σ) become smear-positive (I) and a proportion 1-σ become smear-negative (N).

Of those infected, 1-α become latently infected. Latent infected (L) individuals can progress to TB disease at rate v for reactivation disease. A proportion of latent re-infected ($\alpha(1\text{-}x)$) develop exogenous (reinfection) TB disease (where x defines the level of protection conferred by a previous infection). A proportion of these (σ) become smear-positive (I), 1-σ become smear-negative (N). We assume individuals who recover from smear positive (I) or smear negative (N) TB disease return to the Latent compartment where they are are at identical risk of developing TB disease via reactivation and following reinfection as the rest of individuals in that compartment.

Individuals with prevalent TB disease experience an increased risk of mortality (μ_I or μ_N) that depends on smear status and model stratum (e.g. HIV/ART).

Case detection and treatment

Individuals with smear-positive TB disease are screened at rate γ, a proportion of those that are diagnosed with active disease (true positives) are linked onto care, η. Individuals with smear-negative disease are screened at a lower rate given by $d\gamma$.

A proportion of these individuals (τ) complete treatment and are assumed to return to the latent compartment. Individuals can also naturally recover at rate r, after which they would also return to the latent compartment. Detected cases that do not complete treatment (1-τ) remain in their disease compartment (I or N).

Individuals who do not have active TB disease are screened at a lower rate given by hγ. A proportion of those who are falsely diagnosed with active disease (false positives) are linked onto care, η.

The diagnostic component of the core model includes parameters for the net sensitivity and specificity of different diagnostic algorithms (inputted by the user). Let Se_i and Se_n be the net sensitivities and weighted average based on coverage of different algorithms in a given year for smear positive and smear negative cases, respectively. Similarly, Sp_i and Se_n are the specificities.

Figure 1a - schematic of core TB model

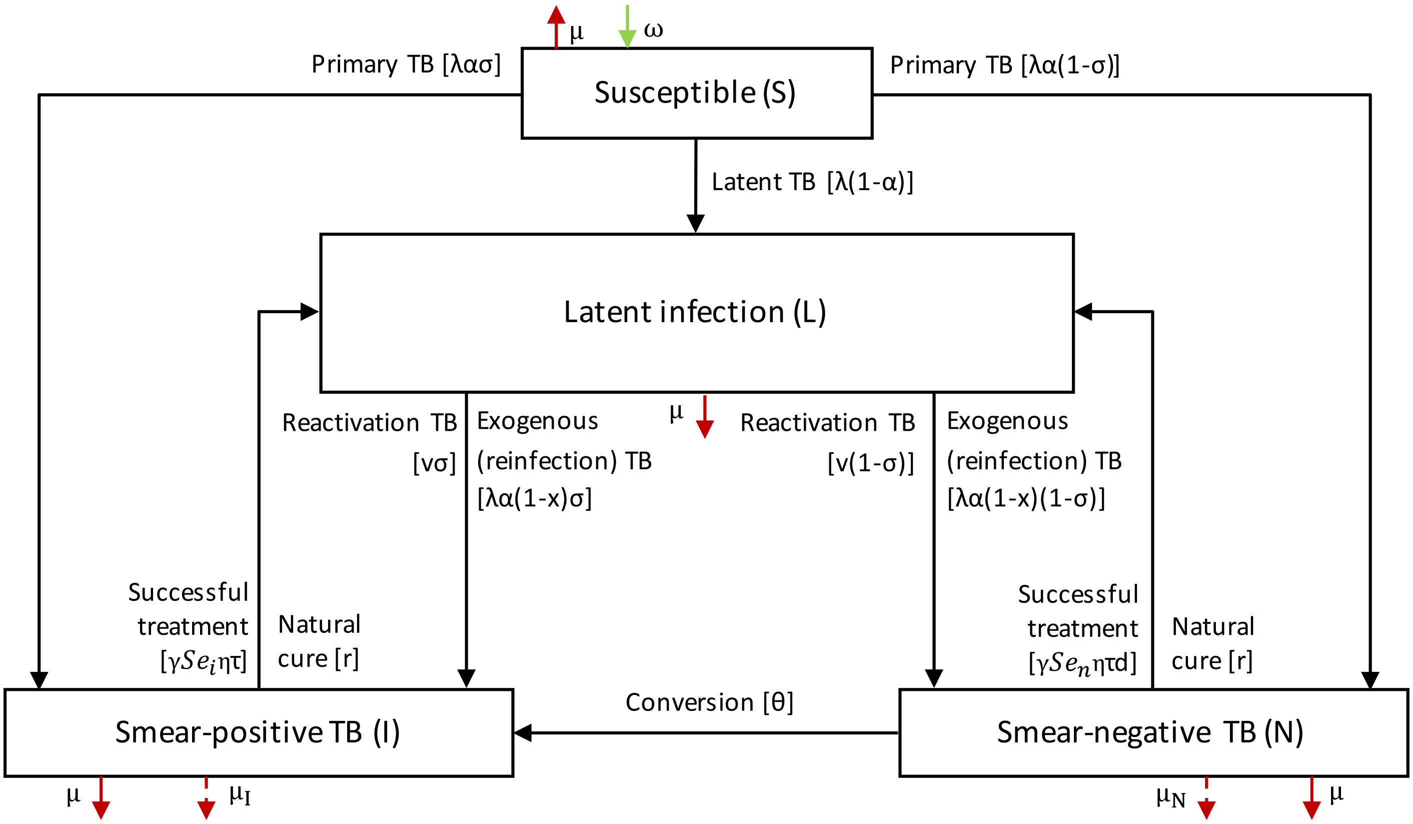

Black arrows represent transitions between TB states, green arrows represent births, red solid arrows represent background deaths, red dashed represent TB deaths

Post-Preventive Therapy Compartment

Interventions that include a preventive therapy component (provision of PT as part of ACF in HIV- population, provision of PT to under 5 year olds) cause a movement of protected individuals to the post-preventive therapy compartment. Individuals in the post-preventive therapy compartment are at risk of re-infection, but not reactivation and experience the protective effect from having a previous infection. The movement of individuals is guided by a *p* factor, which reroutes individuals following infection from S towards the post-PT compartment or following re-infection from L towards post-PT.

Individuals with latent infection, who are screened for TB and are falsely diagnosed with disease (false positives) are assumed to be cleared of their infection if linked onto care and successfully treated and therefore move to the post-PT compartment.

Figure 1b - schematic of core TB model with Post-preventive therapy compartment

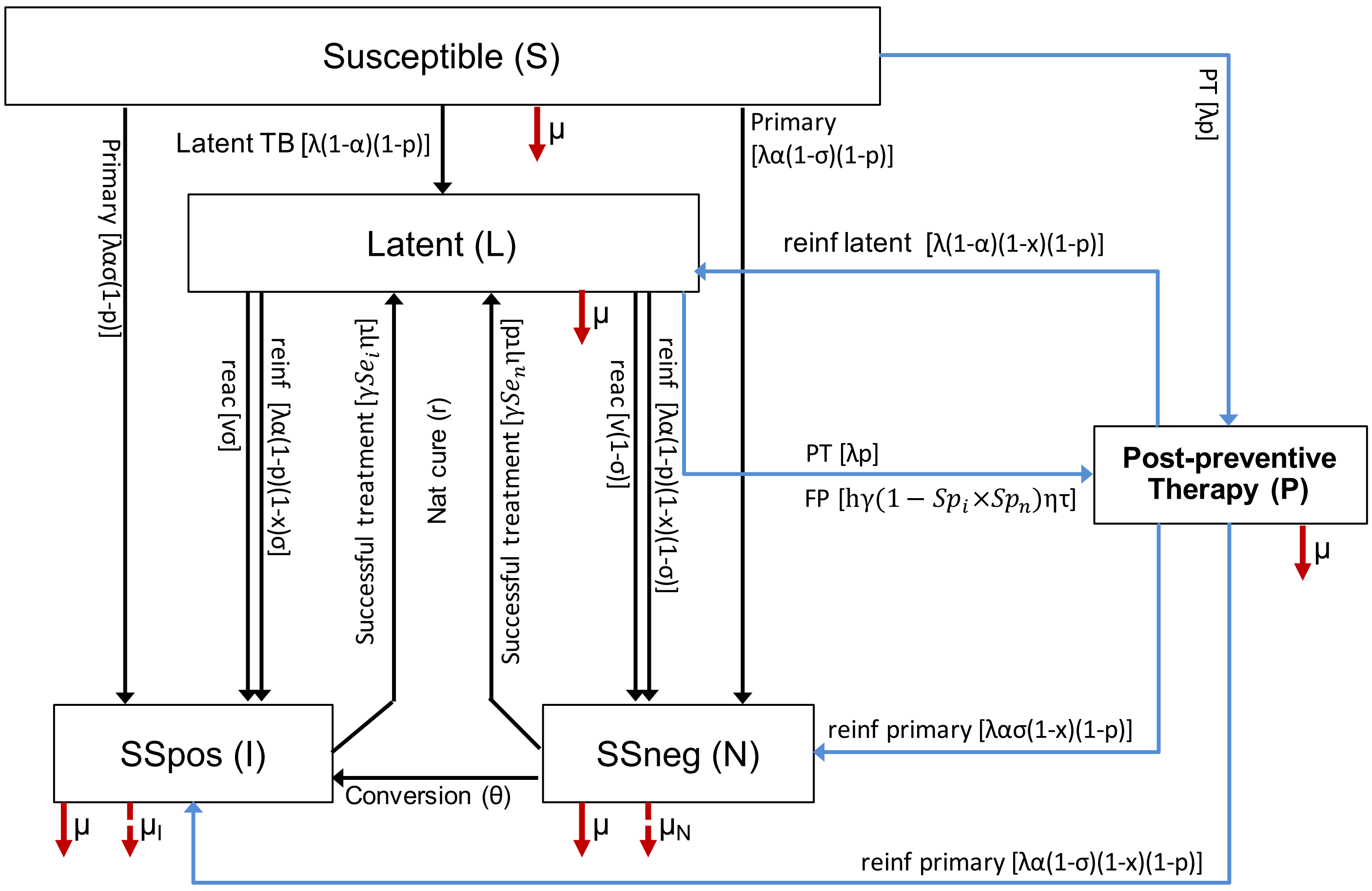

The *p* factor is an age dependent parameter that is calculated for specific interventions and is set to 0 for populations that are not part of the interventions.

For the provision of PT to under 5 years old (see interventions section for more details), the p factor is calculated as follows:

Total number of HH u5s protected due to intervention = N

Number of notified adults*average number of u5 in household*0.304*intervention cov(%)*linkage of INH(%)*complete INH(%)*0.55

Total number of all u5s that would be infected = D

Total u5 $S_{u5\_(t=0)}*\lambda_{(t=-1)} + L_{u5\_(t=0)}*\lambda_{(t=-1)} + P_{u5\_(t=0)}*\lambda_{(t=-1)}$

Proportion of all u5s protected = p

p = N/D

Number of u5s that move from S to post-PT: $S_{u5\_(t=0)}*\lambda_{(t=0)}*p_{(t=0)}$

Number of u5s that progress to disease: $S_{u5\_(t=0)}*\lambda_{(t=0)}*\alpha*(1-p_{(t=0)})$

Number of u5s that progress to latent infection: $S_{u5\_(t=0)}*\lambda_{(t=0)}*(1-\alpha)*(1-p_{(t=0)})$

Therefore, the p factor is applied based on p=N/D (above) only for the 0-4 year old age bin and is set to 0 for all other ages.

3. Age structure

TIME v1.0 is parameterised in 5 year age-bins and assumes homogeneous mixing among all subpopulations and across all ages.

A childhood structure is included in order to capture the epidemiological differences in the paediatric age groups (<15 years old) compared to the adult population (>15 years old).

The parameters affected in paediatric age groups are:

a. Progression to disease (combination of risk of progression to disease and risk of rapid progression)
b. Proportion progressing to smear positive disease
c. Background TB-specific mortality
d. BCG vaccination

Each of these parameters is adjusted using RRs based on review of the literature and are relative to adult TB. An exception to this is BCG vaccination, which is applied to the progression parameters in paediatric age groups as a reduction in risk of progression to disease. Each paediatric age group (0-4, 5-9, 10-14) are adjusted with a different RR, informed by the literature review.

The Epidemiology tab in TIME Impact holds a section for childhood TB. Here, the user is able to make changes to paediatric TB by adjusting the RR for the risk of rapid progression in the 10-14 year old age group. All other RR parameters change proportionately to the adjustment made.

4. Model strata

TIME v1.0 is stratified by HIV/ART status (*number of strata is the same as existing AIM structure*) (1-3), Treatment status (2 strata), MDR status (2 strata). While the TIME model population is also stratified by 1-year age band and sex (as existing Spectrum), TIME is currently parameterised in 5 year age groups.

Treatment History strata

TIME v1.0 is stratified by treatment history, which facilitates modelling the epidemic and interventions related to MDR. There is evidence that the rates of TB disease following reinfection (strong evidence) and reactivation/relapse (less strong) are higher in individuals with a history of TB treatment. However, progression

and infection parameters are currently assumed not to differ by treatment history status, as is convention in most models.

There are two structural effects of stratification by treatment history. Firstly, is that individuals recovering after diagnosis and treatment ($(\mathrm{d})\gamma\mathrm{Se}\eta\tau$) in the treatment naive strata are assumed to move to the Latent compartment in the past treatment strata. This is shown by the solid purple arrows in figure 2. Compartments represent treatment naive individuals (with subscript $_N$, e.g. L_N) or individuals with a past treatment history (with subscript $_P$, e.g. L_P).

To follow the rather strict definition of 'past treatment' (which is usually >2 weeks of exposure to TB drugs), TIME Impact assumes patients started on unsuccessful TB treatment ($(\mathrm{d})\gamma\mathrm{Se}\eta(1-\tau)$) move from I_N to I_P and N_N to N_P. This is shown by the purple dotted lines in figure 2. This makes the simplifying assumptions that all patients who start treatment receive at least two weeks of drugs, and that all smear positive patients receiving unsuccessful treatment remain smear positive. Unsuccessfully treated cases are not counted as additional new incident cases in TIME Impact to reflect the method used by WHO for counting cases. When cases default from treatment, they remain in the prevalent pool. When cases who have defaulted from treatment are rediagnosed, they are counted as retreatment cases as per WHO guidelines.

MDR strata

TIME includes 2 strata based on MDR status, which is the most clinically and policy relevant distinction based on drug sensitivity. Adjustments in the TIME Impact model structure include:

- Acquired resistance

The model allows for acquired resistance. A proportion of those starting first line as initially non-MDR TB disease episode (as per standard DST) are assumed to progress to active disease where MDR is the dominant strain. The underlying process can be that a small number of pre-existing spontaneous MDR mutations were uncovered by first line treatment, or the more classic view that insufficiently effective first line treatment (e.g. through imperfect adherence) creates MDR. In the model, acquiring MDR moves individuals from I_{SEN} or N_{SEN} to the corresponding MDR disease compartment (I_{MDR} or N_{MDR}). The rate of acquiring MDR is determined by parameter ksi (ξ), which is applied directly to a term of non-MDR cases that are diagnosed and started on treatment $(\gamma\mathrm{Se}_i\eta)I_{SEN} + (\mathrm{d}\gamma\mathrm{Se}_n\eta)N_{SEN}$. We assume no MDR is acquired in the absence of treatment. Once the patient has moved to the MDR compartment, we assume they experience the same (MDR specific) linkage to care and second line treatment success as all MDR cases.

Note that ξ is applied directly to the proportion detected and started on treatment ($(d)\gamma\mathrm{Se}\eta$), and that the proportion of successful treatment (τ) is therefore applied to those not acquiring resistance ($(d)\gamma\mathrm{Se}\eta$*(1-ξ)). This allows the model to use values for ξ based on data, but will cause a slight overestimation of treatment failure.

- Initial infection with MDR

There are 2 annual risks of infection (λ= beta*(I+c*N) / P), one for each MDR stratum. The λ term for the MDR strata is adjusted with a relative fitness parameter phi φ, leading to (λ_{MDR}= beta*(I_{MDR}+c*N_{MDR}) / P)*φ.

We assume relative fitness only affects the transmission parameter λ (i.e. risk of first infection and re-infection). We assume it does not affect the protection offered by a previous infection, the rate of rapid progression to TB

disease (α) or the rate of reactivation TB disease (ν). We also assume it does not affect the proportion of active disease that is smear positive (which is already implicitly included in relative fitness parameter).

- Superinfection (defined as reinfection with strain of different drug resistant profile)

In TIME Impact, it is assumed that superinfection occurs. Superinfection can result into either rapid progression to disease, or a latent infection at risk of reactivation in the other MDR stratum.

Superinfections - Rapid Progression: Superinfections that progress to disease ($\lambda_{\text{MDR or Non\_MDR}}\,\alpha(1-\text{x})$) are assumed to move to the disease compartment that matches the drug resistant profile of the superinfection strain, irrespective of the MDR status of the latent or reinfection strain.

Superinfections - Reactivation: We do not explicitly model mixed infections. Superinfections that do not immediately progress to disease are therefore assumed to move to one of the latent strata. In the absence of data and for simplicity, the proportion of non-progressing superinfections that move to the latent MDR compartment (í) is assumed to be based on the relative fitness (φ) parameter, and calculated as $\iota = \frac{\varphi}{1+\varphi}$

In summary, the following rules apply (only terms **in bold** are included in equations, as other terms would keep individuals in same compartment):

1. $\text{Latent}_{\text{sens}}$ + sens reinf → $\text{Latent}_{\text{sens}}$ and $\text{Latent}_{\text{MDR}}$ + MDR reinf → $\text{Latent}_{\text{MDR}}$
2. $\text{Latent}_{\text{sens}}$ + MDR reinf → **í*new infections to $\text{Latent}_{\text{MDR}}$**, (1-í)*new infection stay in $\text{Latent}_{\text{sens}}$
3. $\text{Latent}_{\text{MDR}}$ + sens reinf → í*new infections stay in $\text{Latent}_{\text{MDR}}$, **(1-í)*new infection to $\text{Latent}_{\text{sens}}$**

- Drug sensitivity testing

TIME has an explicit DST parameter, defined as the proportion of all diagnosed cases that receive a DST, by treatment history. This allows inclusion of usually reasonably strong country data, if available. Only if a diagnosed true drug-sensitive case receives a false positive result on DST or a true MDR case receives a true positive results on DST can they receive MDR treatment.

- Treatment success in MDR with first-line drugs

Individuals that don't receive a DST, MDR status remains unknown and they are started on first-line treatment. The literature suggests that MDR cases do experience some treatment success with first-line drugs, though it is lower than for drug sensitive cases (4).

New ($\text{I}_{\text{M\_N}}$ or $\text{N}_{\text{M\_N}}$) MDR cases who get diagnosed with TB disease (non-MDR) and are linked to non-MDR care (η_{S}), but do not receive a DST ($1-\psi_{\text{N}}$) or receive a false negative result on DST ($\boldsymbol{\psi_N(1-\text{Sp}_M)}$) enter the non-MDR care pathway.

A proportion of these are cured with first-line drugs, but at a lower treatment success than is experienced by pan-sensitive cases ($\tau_{\text{S}} \times RR_{\text{N}}$) and enter the latent, previously treated compartment ($\text{L}_{\text{M\_P}}$), where they are at risk for reactivation. Those that fail treatment $\left(1-(\tau_{\text{S}} \times RR_{\text{N}})\right)$ move to active, previously treated, retaining their smear status ($\text{I}_{\text{M\_P}}$ or $\text{N}_{\text{M\_P}}$).

Similarly, previously treated ($\text{I}_{\text{M\_P}}$ or $\text{N}_{\text{M\_P}}$) MDR cases who get diagnosed with TB disease (non-MDR) and are linked to non-MDR TB care (η_{S}), but do not receive a DST ($1-\psi_{\text{P}}$) or receive a false negative results on DST ($\boldsymbol{\psi_N(1-\text{Sp}_M)}$) enter the non-MDR care pathway. They experience a lower treatment success compared to pan-sensitive cases and new MDR cases that are being treated by first-line drugs ($\tau_{\text{S}} \times RR_{\text{P}}$). Those that are

successfully treated enter the latent, previously treated compartment (L_{M_P}) and those that fail treatment remain in the active disease compartment, retaining their smear status $(I_{M\_P}\ or\ N_{M\_P})$.

Interactions between treatment history and MDR strata

There are various interactions between treatment history and MDR status, also reflecting the proportion of cases that receives DST.

New patients with MDR

In the model treatment naïve patients are assumed to only be MDR if they develop disease following a (reactivation of) new or super-infection with an MDR strain. All other MDR TB, be it acquired or initially treated with first line drugs, is assumed to come from the 'previously treated' strata.

Drug sensitivity testing and treatment status

New MDR cases who are diagnosed with TB disease, but do not receive DST or receive a false negative result on DST, are assumed to enter the non-MDR TB care pathway. A proportion of these are assumed to not start treatment (1-linkage to care in the non-MDR TB care pathway) and are assumed to remain in the 'no previous treatment, MDR' disease compartment.

As stated earlier, MDR cases that do not receive a DST enter the non-MDR care pathway where they experience some treatment success with first-line drugs, but less than those with drug-sensitive TB. MDR cases that are successfully treated with first-line drugs are moved to the Latent previously treated compartment. New MDR cases (I_M or N_M) that fail treatment with first-line drugs enter the previously treated active disease compartment, retaining their smear-status.

Disease type and treatment status

The model currently assumes that cases that acquired (or uncovered) MDR during treatment retain their disease type status (smear positive or negative) as they move from the non-MDR to MDR, and from 'no previous treatment' to 'previously treated' stratum.

Figure 2 – Schematic of core TB model with Treatment history stratum

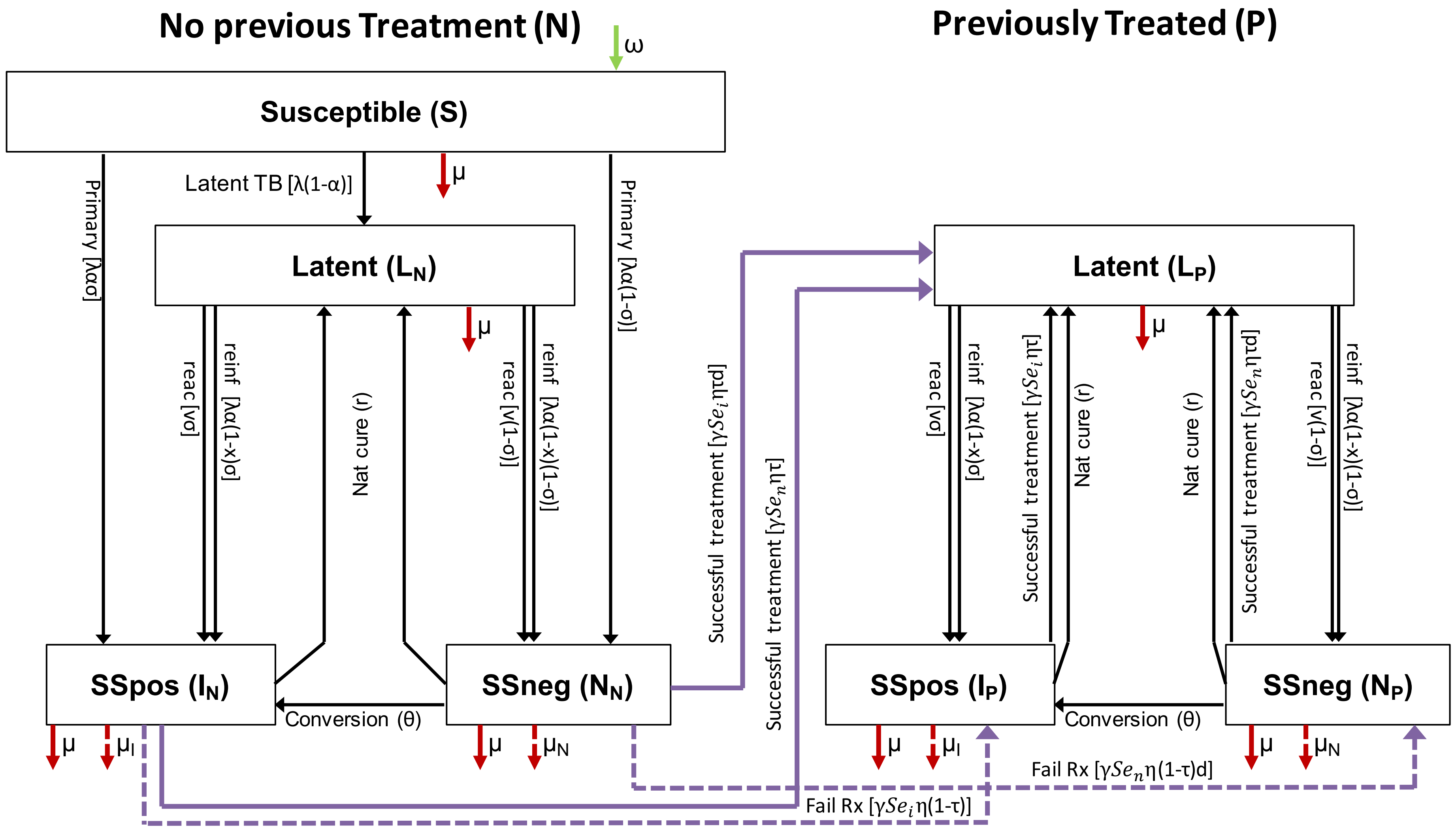

Figure 3. Schematic of MDR stratified core model

Arrows in figure shows all flows between compartments in MDR stratified model. Blue arrows indicate changes in MDR status.

- Compartments or parameters that apply specifically an MDR stratum are indicated by subscripts, e.g. L_S for Latent non-MDR ('sensitive') and L_M for Latent MDR.
- Force of infection with MDR strain has penalty for loss of fitness: λ_{MDR}= beta*(I_{MDR}+c*N_{MDR}) / P)***φ**
- Flow from non-MDR Latent (L_S) to active MDR disease (I_M or N_M) and vice versa represent superinfections that progress rapidly to disease.
- Arrows between Latent (L) compartments represent non-progressing superinfection strains (as described above, flow determined by í)
- Dashed blue lines represent acquired drug resistance (ξ).

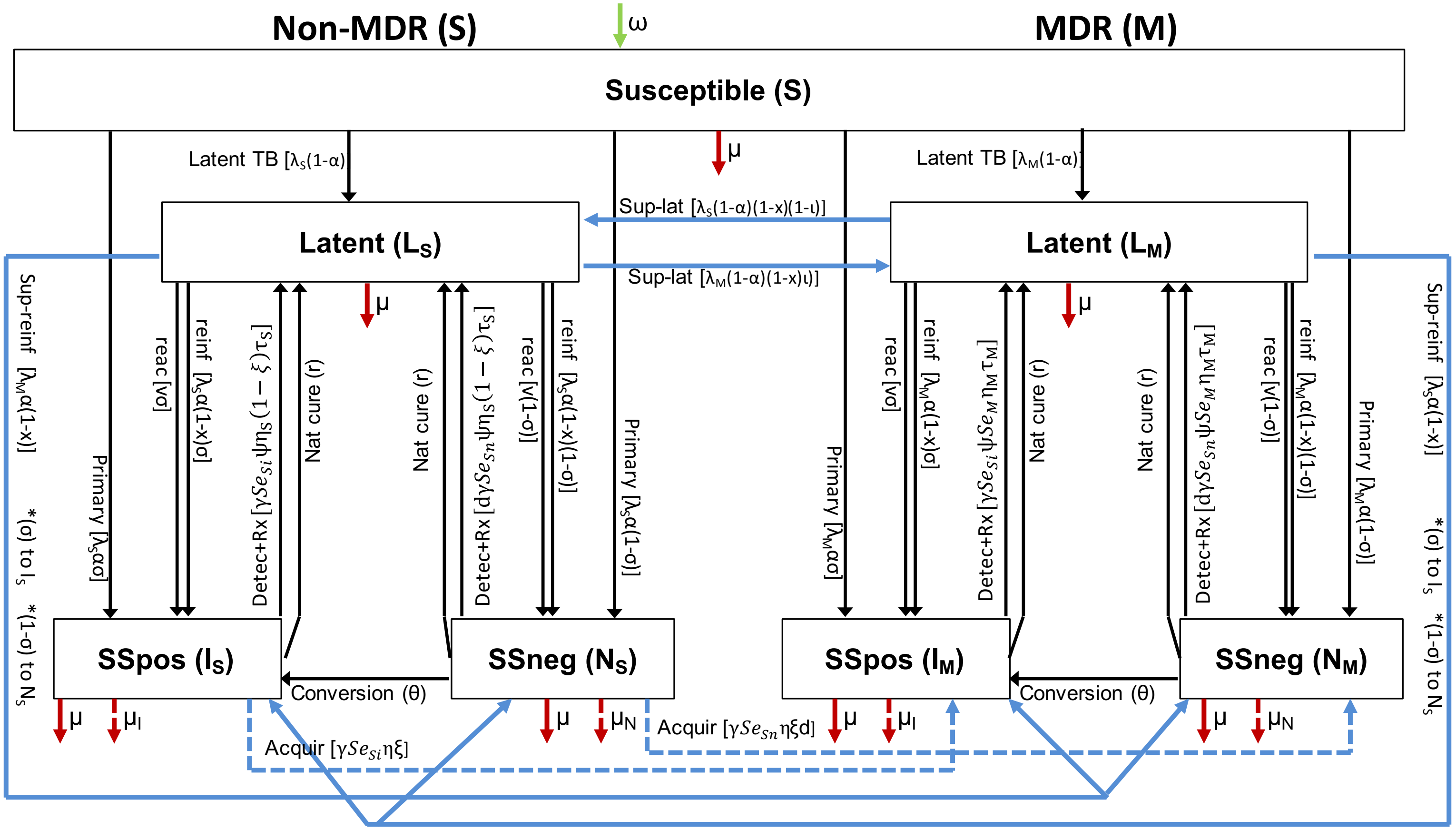

Integration of HIV model

HIV/ART model (AIM)

TIME integrates with the existing Spectrum HIV/ART structure to ensure consistency between HIV and TB models. The following strata are implemented and are used to specify HIV status:

1. HIV negative
2. HIV positive, not on ART
3. HIV positive, on ART for 0-6 months, 7-12 months and on ART for greater than year. The choice of 'on-ART' strata is motivated by mortality data prepared by the Idea consortium, which indicates distinct mortality patterns in these three stages following enrolment on ART.

Each HIV-positive category has seven CD4 stages: CD4 < 50 cells/uL, 50-99, 100-199, 200-249, 250-349, 350-499 and > 500 cells/uL. The following HIV-related parameters vary as a function of CD4 count: progression to lower CD4 counts, HIV-specific mortality, probability of initiating ART, and HIV infectiousness. Spectrum's ART categories are also structured by the same seven CD4 categories, but they are used only to keep track of CD4 at ART initiation. Spectrum does not model recovery of CD4 count following ART. Many of the HIV parameters are also stratified by sex and age. These parameters are then represented in a table structured by sex and 10-year age bins.

The following equations describe the demographic HIV model in Spectrum. **S** denotes individuals susceptible to HIV, **I** individuals who are HIV positive but not yet receiving ART and **A** HIV positive individuals receiving ART. Each of the variables is structured by time (**t**) and age (**a**). **I** is further subdivided by CD4 category (**c**=1...7), whereas **A** is subdivided by CD4 category (**c**=1...7) and duration of ART (**d**=1,2,3).

The following parameters are used to describe the demographical and HIV processes:
μ(a) – age-specific background mortality,
H(t,a) time and age-specific number of new infections from Spectrum,
H(t,a,c) time, age and CD4 specific number of new infections from Spectrum,
β(a,c) – age and CD4 specific HIV mortality,
β(a,c,d) – age, CD4 and ART duration specific HIV mortality,
ϕ(a,c) age and CD4-specific progression rate,
a(t,a,c) time, age and CD4 specific ART initiation numbers from Spectrum,
σ(d) specifies movement to subsequent ART categories.

For **d**=1 and **d**=2 **σ**(d)=2, modelling an average duration of 6 months. Each parameter can also vary by sex (not shown in equations).

HIV model equations

Susceptible (S)

$$\frac{dS(t,a)}{dt} + \frac{dS(t,a)}{da} = -\mu(a)S(t,a) - H(t,a)$$

HIV+ not on ART (I)

$$\frac{dI(t,a,c)}{dt} + \frac{dI(t,a,c)}{da} = -\big(\mu(a) + \beta(a,c) + \phi(a,c)\big)I(t,a,c) + \phi(a,c-1)I(t,a,c-1) + H(t,a,c) - a(t,a,c)$$

HIV+ on ART (A)

$$\frac{dA(t,a,c,d)}{dt} + \frac{dA(t,a,c,d)}{da} = -(\mu(a) + \beta(a,c,d) + \sigma(d))A(t,a,c) + \sigma(d-1)A(t,a,c,d) + a(t,a,c)$$

Integration of HIV model with TB strata/states

New HIV infections and ART initiations:

AIM calculates the age/sex group specific number of incident HIV infections that occur during the time step, and distributes these evenly across the HIV negative TB states and strata, weighted by size of the population in that state/stratum. It does not take into account:

- Risk of HIV infection during TB disease. This may not be highly relevant given the size of the compartment (<1% of population in almost every country)
- TB as an indication of ART eligibility. HIV positive individuals with TB disease are eligible for ART, regardless of CD4 count; therefore, their likelihood of being initiated on ART is higher compared to other HIV positive individuals in their respective CD4 stratum. However, TIME Impact includes an intervention to reflect screening of HIV in active TB cases and linking HIV+ into ART care (see Interventions chapter).

HIV progression and TB states

If an HIV positive individual moves between TB states, they automatically transfer to the corresponding CD4/ART category within that new TB state, and continue with the same HIV progression.

HIV/TB mortality

All HIV positive categories experience a higher background mortality. The value for HIV specific mortality is drawn from AIM, and is CD4 dependent. It would be inappropriate to apply the default values, which include deaths due to TB, to non-TB disease states (Susceptible and Latent) in the TB model, as these populations are not at risk of dying from the disease. To adjust for this, the default AIM mortality rate is reduced by 25% (global estimate of all HIV deaths that are due to TB, (5)).

ART allocation

Total number of new ART allocations (which comes from AIM per CD4 category) gets divided in proportion to population size and mortality of the different compartments.

That is, start_art(x)=(W1(x)+W2(x))/2*new_art

Where x is the state label and W1 is the size of x relative to all eligible for ART and W2 the proportion of all mortality among those eligible that happen in x.

Impact of HIV and ART on TB natural history parameters

All natural history TB parameters can be modified by HIV status, though by default, smear conversion rate and MDR fitness and acquisition parameters are set as the same.

The parameters for rapid progression to TB, the reactivation rate and the protection offered by prior infection are dependent on HIV status and CD4 category. Following a model by Williams et al, the user specifies two relative risks:

RR1: the initial change in risk attributable to HIV infection (i.e. in the CD4>500 category).
RR2: the change in risk with each 100-cells/uL change in the CD4 count.

The final parameter value is calculated as P(HIV-) x RR1 x RR2 ^{CD4}. The value ^{CD4} is taken as (500 – midpoint value for that CD4 strata)/100. E.g. for the 350-500 strata, the mid-point is 425, so the value is 0.75.

The impact of ART is to reduce the difference between the parameter value for that CD4 strata and the HIV negative value for that parameter. The effect increases with time spent on ART.

Default values for these parameters and references can be found in the table 2.

Screening for disease

Diseased individuals enter the screening population at a rate of γ and follow a diagnostic algorithm with a net sensitivity of Se_{S_i} for smear positive disease and Se_{S_n} for smear negative disease. A relative screening rate of d is applied to smear negative TB. A proportion ψ_N (for treatment naïve) or ψ_P (for previously treated) of cases will receive a DST with a test sensitivity of Se_M and a specificity of Sp_M. A proportion η_S (for drug sensitive TB) and η_M (for MDR TB) are linked onto first or second line care, respectively.

Individuals with pan-sensitive TB disease, are therefore diagnosed and linked onto first-line care at a rate of $\gamma\mathrm{Se}_{S\_i}\psi_N\mathrm{Sp}_M\eta_S$ for those that receive a DST or $\gamma\mathrm{Se}_{S_i}(1-\psi_N)\eta_S$ for those that do not receive a DST.

A proportion of those who are treated successfully τ_S enter the latent, previously treated compartment. Those that fail treatment $(1-\tau_S)$ either move to the active disease previously treated compartment (if they were treatment naïve) or remain in the active disease previously treated compartment if they were already previously treated, retaining their smear status. A proportion of cases with drug sensitive TB disease that are linked onto first-line care ξ develop MDR-TB and are moved to the previously treated active MDR TB compartment, retaining their smear status.

Individuals with true drug sensitive TB may receive a false positive results on DST and are therefore falsely linked onto second line care $\gamma\mathrm{Se}_{S\_i}\psi(1-\mathrm{Sp}_M)\eta_M$. It is assumed that these individuals experience the same treatment success probability as those with MDR TB who are linked onto second line care τ_M.

Individuals with MDR TB disease can only be linked onto appropriate second line care if they receive a DST <u>and</u> if the results are true positive. Individuals with false negative result on DST are linked onto first-line care, where they experience a reduced treatment success probability: $\gamma\mathrm{Se}_{S\_i}\psi_N(1-\mathrm{Se}_M)\eta_S(\tau_S\times\mathrm{RR}_N)$ for treatment naïve individuals and $\gamma\mathrm{Se}_{S\_i}\psi_P(1-\mathrm{Se}_M)\eta_S(\tau_S\times\mathrm{RR}_P)$ for previously treated individuals.

<u>Note:</u> Smear positive or negative cases that are not picked up by the net sensitivity of the algorithm (i.e. $1-\mathrm{Se}_i$ for smear positive and $1-\mathrm{Se}_n$ for smear negative) are undetected (false negative) cases and remain in their respective disease compartments.

Individuals from the susceptible compartment that enter the screening population at a rate of $\mathrm{h}\gamma$ can be false positive cases $(1-(\mathrm{Sp}_i\times\mathrm{Sp}_n))$. These individuals remain in the susceptible compartment, ignoring any protective effects that may be offered while the individuals are on treatment.

Individuals from the latent compart also enter the screening population at a rate of $\mathrm{h}\gamma$. However, those that have been screened for TB, are false positive, have been linked onto first-line care and have completed treatment $(\mathrm{h}\gamma(1-(\mathrm{Sp}_i\times\mathrm{Sp}_n))\eta_S\tau_S)$ are considered cured of the Mtb latent infection and move to the Post-preventive therapy box,

where they are at risk of reinfection but not reactivation. Note that this does not apply to individuals with latent MDR infection.

5. Full model equations (excluding HIV strata and interventions)

Table 1. Legend for model parameters used in equations

**Epidemiology/natural history**
β, effective contact rate
*c, r*elative infectiousness of smear-negative cases
λ, annual rate of infection = β(I+c*N) / T (I=SSpos, N=SSneg, T=total population)
α, proportion of infections developing primary TB
v, rate at which latently infected individuals develop active TB disease
σ, proportion of cases developing smear positive TB disease
x, protection provided by prior infection*
r, self cure rate
θ, rate of conversion from SSneg to SSpos disease (N to I)
μ_I, TB disease mortality rate (SSneg)
μ_N, TB disease mortality rate (SSpos)
**Care and control**
η, proportion linked into care
γ, screening rate
d, relative screening rate of smear-negative cases
τ, treatment success, by MDR status
h, relative screening rate of individuals without active TB disease (susceptible and latent)
p, protection from preventive therapy
Se, sensitivity of screening, by smear status and MDR status
Sp, specificity of screening, by smear status and MDR status
**MDR specific parameters**
ξ, % acquiring MDR Risk acquiring MDR under treatment (% per treatment episode)
φ, relative fitness of MDR strains
Ί, proportion of non-progressing superinfections that move to the latent MDR compartment = $\frac{\varphi}{1+\varphi}$
ψ, proportion of diagnosed MDR TB cases that receive a DST (separated by treatment history status ψ_N & ψ_P)

Model equations

Susceptible population (S)

$$\frac{dS}{dt} = +\omega - (\lambda_S + \lambda_M + \mu)S$$

<u>Non-MDR, drug susceptible strains</u>

Latent treatment naïve

$$\frac{dL_{S\_N}}{dt} = -\left(\nu + \lambda_S(\alpha(1-x)(1-p)+p) + (\lambda_M\alpha(1-x) + \lambda_M(1-\alpha)(1-x)\acute{\iota} + \mu) + h\gamma\left(1-(Sp_i \times Sp_n)\right)\eta_S\tau_S\right)L_{S\_N}$$
$$+ \lambda_S(1-\alpha)(1-p)S + rI_{S_N} + rN_{S_N}$$
$$+\lambda_S(1-\alpha)(1-x)(1-\acute{\iota})L_{M\_N} + \lambda_S(1-\alpha)(1-x)(1-p)P_{S\_N}$$

Latent previously treated

$$\frac{dL_{S\_P}}{dt} = -\left(\nu + \lambda_S(\alpha(1-x)(1-p)+p) + (\lambda_M\alpha(1-x) + \lambda_M(1-\alpha)(1-x)\acute{\iota} + \mu) + h\gamma\left(1-(Sp_i \times Sp_n)\right)\eta_S\tau_S\right)L_{S\_P}$$
$$+ \left(r + \gamma Se_{S\_i}\psi_P Sp_M\eta_S(1-\xi)\tau_S + \gamma Se_{S\_i}(1-\psi_P)\eta_S(1-\xi)\tau_S + \gamma Se_{S\_i}\psi_P(1-Sp_M)\eta_M\tau_M\right)I_{S\_P}$$
$$+\left(r + d\gamma Se_{S\_n}\psi_P Sp_M\eta_S(1-\xi)\tau_S + d\gamma Se_{S\_n}(1-\psi_P)\eta_S(1-\xi)\tau_S + d\gamma Se_{S\_n}\psi_P(1-Sp_M)\eta_M\tau_M\right)N_{S\_P}$$
$$+\lambda_S(1-\alpha)(1-x)(1-\acute{\iota})L_{M\_P}$$
$$+ \left(\gamma Se_{S\_i}\psi_N Sp_M\eta_S(1-\xi)\tau_S + \gamma Se_{S\_i}(1-\psi_N)\eta_S(1-\xi)\tau_S + \gamma Se_{S\_i}\psi_N(1-Sp_M)\eta_M\tau_M\right)I_{S\_N}$$
$$+\left(d\gamma Se_{S\_n}\psi_N Sp_M\eta_S(1-\xi)\tau_S + d\gamma Se_{S\_n}(1-\psi_N)\eta_S(1-\xi)\tau_S + d\gamma Se_{S\_n}\psi_N(1-Sp_M)\eta_M\tau_M\right)N_{S\_N}$$
$$+ \lambda_S(1-\alpha)(1-x)(1-p)P_{S\_P}$$

Active Smear Positive treatment naïve

$$\frac{dI_{S\_N}}{dt} = \lambda_S\alpha\sigma(1-p)S + \left(\nu\sigma + \lambda_S\alpha\sigma(1-x)(1-p)\right)L_{S_N} + \lambda_S\alpha\sigma(1-x)(1-p)P_{S\_N} + \theta N_{S\_N} + \lambda_S\alpha(1-x)\sigma L_{M\_N}$$
$$-(\mu + \mu_I + r + \gamma Se_{S\_i}\psi_N Sp_M\eta_S + \gamma Se_{S\_i}(1-\psi_N)\eta_S + \gamma Se_{S\_i}\psi_N(1-Sp_M)\eta_M)I_{S\_N}$$

Active Smear Positive previously treated

$$\frac{dI_{S_P}}{dt} = \left(\nu\sigma + \lambda_S\alpha\sigma(1-x)(1-p)\right)L_{S_P} + \lambda_S\alpha\sigma(1-x)(1-p)P_{S_P} + \theta N_{S_P} + \lambda_S\alpha(1-x)\sigma L_{M_P}$$
$$+ (\gamma Se_{S_i}\psi_N Sp_M\eta_S(1-\xi)(1-\tau_S) + \gamma Se_{S\_i}(1-\psi_N)\eta_S(1-\xi)(1-\tau_S) + \gamma Se_{S_i}\psi_N(1-Sp_M)\eta_M(1-\tau_M))I_{S\_N}$$
$$-\left(\mu + \mu_I + r + \gamma Se_{S\_i}\psi_P Sp_M\eta_S(1-\xi)\tau_S + \gamma Se_{S_i}(1-\psi_P)\right)\eta_S(1-\xi)\tau_S + \gamma Se_{S_i}\psi_P(1-Sp_M)\eta_M\tau_M$$
$$+ \gamma Se_{S_i}\psi_P Sp_M\eta_S\xi + \gamma Se_{S\_i}(1-\psi_P)\eta_S\xi)I_{S\_P}$$

Active Smear Negative treatment naïve

$$\frac{dN_{S_N}}{dt} = \lambda_S\alpha(1-\sigma)(1-p)S + \left(\nu(1-\sigma) + \lambda_S\alpha(1-\sigma)(1-x)(1-p)\right)L_{S_N}$$
$$+\lambda_S\alpha(1-\sigma)(1-x)(1-p)P_{S_N} + \lambda_S\alpha(1-x)(1-\sigma)L_{M_N}$$
$$-(\theta + \mu + \mu_N + r + d\gamma Se_{S_n}\psi_N Sp_M\eta_S + d\gamma Se_{S\_n}(1-\psi_N)\eta_S + d\gamma Se_{S\_n}\psi_N(1-Sp_M)\eta_M)N_{S\_N}$$

Active Smear Negative previously treated

$$\frac{dN_{S_P}}{dt} = \big(\nu(1-\sigma) + \lambda_S\alpha(1-\sigma)(1-x)(1-p)\big)L_{S_P} + \lambda_S\alpha(1-\sigma)(1-x)(1-p)P_{S\_P} + \lambda_S\alpha(1-x)(1-\sigma)L_{M\_P}$$
$$+ \big(d\gamma Se_{S_n}\psi_N Sp_M\eta_S(1-\xi)(1-\tau_S) + d\gamma Se_{S_n}(1-\psi_N)\eta_S(1-\xi)(1-\tau_S)$$
$$+ d\gamma Se_{S\_n}\psi_N(1-Sp_M)\eta_M(1-\tau_M)\big)N_{S\_N}$$
$$-\big(\theta + \mu + \mu_N + r + d\gamma Se_{S_n}\psi_P Sp_M\eta_S(1-\xi)\tau_S + d\gamma Se_{S_n}(1-\psi_P)\eta_S(1-\xi)\tau_S + d\gamma Se_{S_i}\psi_P(1-Sp_M)\eta_M\tau_M$$
$$+ d\gamma Se_{S\_n}\psi_P Sp_M\eta_S\xi + d\gamma Se_{S\_n}(1-\psi_P)\eta_S\xi\big)N_{S\_P}$$

Post-preventive therapy, treatment naïve

$$\frac{dP_{S\_N}}{dt} = \lambda_S pS + \big(\lambda_S p + h\gamma\big(1-(Sp_i \times Sp_n)\big)\eta_S\tau_S\big)L_{S\_N} - \big(\lambda_S(1-x)(1-p) + \lambda_M\alpha(1-x) + \lambda_M(1-\alpha)(1-x)\acute{\iota}$$
$$+ \mu\big)P_{S\_N}$$

Post-preventive therapy, previously treated

$$\frac{dP_{S\_P}}{dt} = \big(\lambda_S p + h\gamma\big(1-(Sp_i \times Sp_n)\big)\eta_S\tau_S\big)L_{S\_P} - \big(\lambda_S(1-x)(1-p) + \lambda_M\alpha(1-x) + \lambda_M(1-\alpha)(1-x)\acute{\iota} + \mu\big)P_{S\_P}$$

<u>MDR strains</u>

Latent treatment naïve

$$\frac{dL_{M\_N}}{dt} = -\big(\nu + \lambda_S\alpha(1-x) + (\lambda_M\alpha(1-x) + \lambda_S(1-\alpha)(1-x)(1-\acute{\iota}) + \mu)\big)L_{M\_N}$$
$$+ \lambda_M(1-\alpha)S + rI_{M\_N} + rN_{M\_N} + (\lambda_M(1-\alpha)(1-x)\acute{\iota})L_{S_N} + (\lambda_M\alpha(1-x) + \lambda_M(1-\alpha)(1-x)\acute{\iota})\,P_{S\_N}$$

Latent previously treated

$$\frac{dL_{M\_P}}{dt} = -\big(\nu + \lambda_S\alpha(1-x) + (\lambda_M\alpha(1-x) + \lambda_S(1-\alpha)(1-x)(1-\acute{\iota}) + \mu)\big)L_{M\_P}$$
$$+ \Big(r + \gamma Se_{S\_i}\psi_P Se_M\eta_M\tau_M + \gamma Se_{S\_i}(1-\psi_P)\eta_S(\tau_S \times RR_P) + \gamma Se_{S\_i}\psi_P(1-Se_M)\eta_S(\tau_S \times RR_P)\Big)\,I_{M\_P}$$
$$+ \Big(r + d\gamma Se_{S\_n}\psi_P Se_M\eta_M\tau_M + d\gamma Se_{S\_n}(1-\psi_P)\eta_S(\tau_S \times RR_P) + d\gamma Se_{S\_n}\psi_P(1-Se_M)\eta_S(\tau_S \times RR_P)\Big)\,N_{M\_P}$$
$$+(\lambda_M(1-\alpha)(1-x)\acute{\iota})L_{S\_P}$$
$$+ \Big(\gamma Se_{S\_i}\psi_N Se_M\eta_M\tau_M + \gamma Se_{S\_i}(1-\psi_N)\eta_S(\tau_S \times RR_N) + \gamma Se_{S\_i}\psi_N(1-Se_M)\eta_S(\tau_S \times RR_N)\Big)\,I_{M\_N}$$
$$+ (d\gamma Se_{S_n}\psi_N Se_M\eta_M\tau_M + d\gamma Se_{S_n}(1-\psi_N)\eta_S(\tau_S \times RR_N) + d\gamma Se_{S_n}\psi_N(1-Se_M)\eta_S(\tau_S \times RR_N))N_{M_N}$$
$$+\lambda_M\alpha(1-x) + \lambda_M(1-\alpha)(1-x)\acute{\iota} + \mu)P_{S\_P}$$

Active Smear Positive treatment naïve

$$\frac{dI_{M\_N}}{dt} = (\lambda_M\alpha\sigma)S + (\nu\sigma + \lambda_M\alpha\sigma(1-x))L_{M\_N} + \theta N_{M\_N} + \lambda_M\alpha(1-x)\sigma L_{S\_N}$$
$$-(\mu + \mu_I + r + \gamma Se_{S\_i}\psi_N Se_M\eta_M + \gamma Se_{S\_i}(1-\psi_N)\eta_S + \gamma Se_{S\_i}\psi_N(1-Se_M)\eta_S)I_{M\_N}$$

Active Smear Positive previously treated

$$\frac{dI_{M\_P}}{dt} = \big(\nu\sigma + \lambda_M\alpha\sigma(1-x)\big)L_{M_P} + \theta N_{M_P} + \lambda_M\alpha(1-x)\sigma L_{S_P}$$
$$+(\gamma Se_{S\_i}\psi_N Se_M\eta_M(1-\tau_M) + \gamma Se_{S_i}(1-\psi_N)\eta_S\big(1-(\tau_S\times RR_N)\big) + \gamma Se_{S\_i}\psi_N(1-Se_M)\eta_S\big(1-(\tau_S\times RR_N)\big))\,I_{M\_N}$$
$$+(\gamma Se_{S_i}\psi_N Sp_M\eta_S\xi + \gamma Se_{S_i}(1-\psi_N)\eta_S\xi)I_{S_N} + (\gamma Se_{S_i}\psi_P Sp_M\eta_S\xi + \gamma Se_{S\_i}(1-\psi_P)\eta_S\xi)I_{S\_P}$$
$$-(\mu + \mu_I + r + \gamma Se_{S\_i}\psi_P Se_M\eta_M\tau_M + \gamma Se_{S\_i}(1-\psi_P)\eta_S(\tau_S\times RR_P) + \gamma Se_{S\_i}\psi_P(1-Se_M)\eta_S(\tau_S\times RR_P))I_{M\_P}$$

Active Smear Negative treatment naïve

$$\frac{dN_{M\_N}}{dt} = (\lambda_M\alpha(1-\sigma))S + (\nu(1-\sigma) + \lambda_M\alpha(1-\sigma)(1-x))L_{M\_N} + \lambda_M\alpha(1-x)(1-\sigma)L_{S\_N}$$
$$-(\theta + \mu + \mu_N + r + d\gamma Se_{S_n}\psi_N Se_M\eta_M + d\gamma Se_{S_n}(1-\psi_N)\eta_S + d\gamma Se_{S_n}\psi_N(1-Se_M)\eta_S)N_{M\_N}$$

Active Smear Negative previously treated

$$\frac{dN_{M\_P}}{dt} = \big(\nu(1-\sigma) + \lambda_M\alpha(1-\sigma)(1-x)\big)L_{M_P} + \lambda_M\alpha(1-x)(1-\sigma)L_{S_P}$$
$$+(d\gamma Se_{S_n}\psi_N Se_M\eta_M(1-\tau_M) + d\gamma Se_{S_n}(1-\psi_N)\eta_S\big(1-(\tau_S\times RR_N)\big)$$
$$+ d\gamma Se_{S_n}\psi_N(1-Se_M)\eta_S\big(1-(\tau_S\times RR_N)\big))N_{M\_N}$$
$$+(d\gamma Se_{S_n}\psi_N Sp_M\eta_S\xi + d\gamma Se_{S\_n}(1-\psi_N)\eta_S\xi)N_{S\_N} + (\gamma Se_{S\_n}\psi_P Sp_M\eta_S\xi + d\gamma Se_{S\_n}(1-\psi_P)\eta_S\xi)N_{S\_P}$$
$$-(\theta + \mu + \mu_N + r + d\gamma Se_{S_n}\psi_N Se_M\eta_M\tau_M + d\gamma Se_{S\_n}(1-\psi_P)\eta_S(\tau_S\times RR_P)$$
$$+ d\gamma Se_{S\_n}\psi_P(1-Se_M)\eta_S(\tau_S\times RR_P))N_{M\_P}$$

6. Model parameters

Default value and ranges for TIME Impact model parameters

Table 2 below shows the current default parameter values and recommended ranges. The sources currently reflect values and ranges used in other models. As time allows, these will be updated with more empirical sources. The values in the grey boxes are the values currently used in TIME.

Table 2. Default values for parameters

	Point estimate	Lower bound	Upper bound	Source	Notes
Progression to TB					
HIV-					
Develop Primary TB (%)	11.5	9	14	(6)	
	14	5	25	(7)	
		8	15	(8)	
	11.5	8	15	(6) (8)	
Reactivation rate (%/year)	0.1	0.03	0.24	(6)	
	0.11	0.05	0.25	(7)	
		0.01	0.1	(8)	
	0.1	0.01	0.25	(6) (7) (8)	
Protection provided by prior infection (%)	65	37	85	(6)	
	72	60	100	(7)	

		40	90	(8)	
	65	37	90	(6) (8)	
Risk Ratio parameter 1					
Develop primary TB (%)	2.6	2.11	3.2	(9)	
Reactivation rate (%/year)	2.6	2.11	3.2	(9)	
Protection provided by prior infection (%)	0.8	0.6	1	(6)	
Risk Ratio parameter 2					
Develop primary TB (%)	1.36	1.3	1.42	(10)	
Reactivation rate (%/year)	1.36	1.3	1.42	(10)	
Protection provided by prior infection (%)	-1.3	-2	-1	(6)	
Smear status					
HIV-					
Cases developing SSpos TB (%)	62	42	80	(6)	
		40	50	(11)	
		40	80	(8)	
	45	40	50	(11)	Point estimate taken as midpoint
Relative infectiousness SSneg TB (%)	22	16	32	(12)	
	22	10	30	(7)	

	22	12	37	(6)	
	22	10	37	(6) (7)	
Smear conversion rate (%/year)	1.5	1	2.3	(6)	
	1.5	0.7	2	(8)	
	2	1	3	(7)	
	1.5	0.7	3	(7) (8)	
HIV+ (CD4>500)					
Cases developing SSpos TB (%)	45	23	68	(6)	
		24	61	(13)	
	32.7	21.9	42.5	(6) (11)	Assuming proportion between HIV- and HIV+ from (6)
Relative infectiousness SSneg (%)	22	10	37	(6) (7)	Assuming same as HIV-
Smear conversion rate (%/year)	2.25	1.5	3	Default	
Recovery					
HIV-					
Self cure rate (%/year)	20	15	25	(6)	
		10	25	(8)	
	20	10	25	(6) (8)	
HIV+ (CD4>500)					

Self cure rate (%/year)	10	6	16	(6)	
TB Mortality					
HIV-					
TB morality rate (SSpos) (%/year)	30	21	41	(6)	
	30	20	40	(8)	
	30	20	41	(6) (8)	
TB mortality rate (SSneg) (%/year)	21	18	25	(6) (8)	
HIV+ (CD4>500)					
TB mortality rate (SSpos) (%/year)	60	40	82	(6) (8)	Assume double HIV-
TB mortality rate (SSneg) (%/year)	42	36	50	(6) (8)	Assume double HIV-
MDR					
HIV-					
Relative fitness of MDR strains (%)	73	58	85	(6)	
Risk acquiring MDR under treatment (% per treatment episode)	1.4	1	1.7	(14)	
Treatment success when using FL for MDR treatment naive	0.61	0.53	0.70	(4)	
Treatment success when using FL for MDR previously treated	0.45	0.35	0.58	(4)	
HIV+ (CD4>500)					

Relative fitness of MDR strains (%)	73	58	85	(6)	Assume same as HIV-
Risk acquiring MDR under treatment (% per treatment episode)	1.4	1	1.7	(14)	Assume same as HIV-
Treatment success when using FL for MDR treatment naive	0.61	0.53	0.70	(4)	
Treatment success when using FL for MDR previously treated	0.45	0.35	0.58	(4)	
Protection offered by ART % reduction of impact of HIV					
Progression					
ART <6m (%)	20.4	15.75	26.83	(15)	Assume linear interpolation between 0 at 0 months and 70 at 12 months
ART 7m-12m (%)	55.4	42.75	72.83	(15)	Assume linear interpolation between 0 at 0 months and 70 at 12 months
ART >1 year (%)	70	54	92	(15)	
	81	62	91	(16)	
	70	54	92	(15)	Assume plateau after 12 months
Mortality					
ART <6m (%)	23.2	10.7	27.7	(15)	Assume linear interpolation between 0 at 0 months and 79.5 at 12 months
ART 7m-12m (%)	62.9	50.7	75.2	(15)	Assume linear interpolation between 0 at 0 months and 79.5 at 12 months
ART >1 year (%)		64	95	(15)	

	79.5	64	95	(15)	Point estimate taken as midpoint between ranges provided; assuming plateau after 12 months.
Care and control					
Effective contact rate (n/year)	22	0	∞		Not bounded a priori

Table 3, below, shows the parameters for paediatric TB. The RRs shown are relative to adult parameters.

Table 3. Default RR and risks for paediatric TB.

**Parameter and age group**	**RR**	**Paediatric risk**	**Ref**
Risk of rapid progression			(6, 8, 17) expert opinion
10-14 years old	0.47	5.4%	
5-9 years old	1.18	13.6%	
0-4 years old	2.22	25.5%	
Risk of smear positivity			(11, 18, 19)
10-14 years old	0.67	30.2%	
5-9 years old	0.34	15.3%	
0-4 years old	0.02	0.7%	
Mortality rate, Smear positive			Expert opinion
10-14 years old	1	30	
5-9 years old	1	30	
0-4 years old	2	60	
Mortality rate, Smear negative			Expert opinion
10-14 years old	1	21	
5-9 years old	1	21	
0-4 years old	2	42	

Another important parameter in paediatric TB is protection offered by BCG in vaccinated children. Overall (extrapulmonary and pulmonary) weighted average of BCG protection is 56.24% (39%-72%) (20) and is applied to the progression parameters of paediatric age groups. BCG can be turned on all via a toggle switch based on country-specific policy.

7. Interventions

Methods
There are 2 ways of implementing interventions in TIME. Firstly, one can use the intervention matrix by providing intervention information such as impact size, sensitivity of screening algorithms (if needed) and time-dependent parameters (eg. Coverage). Secondly, the user can formulate a custom intervention directly by changing the Care and Control parameters.

Intervention Matrix
TIME offers an intervention matrix that explicitly shows which model parameters are changed by each intervention, and by how much. The population wide impact depends on this intervention matrix, the proportion of the population that is covered by the intervention at a given time. The interventions currently included in TIME are listed and their related assumptions are listed below.

Increased Case Detection (non-MDR)
This increases the rate at which non-MDR TB cases are diagnosed. The user can specify by what % the diagnostic rate is changed (currently limits are set at -100% to infinity). The coverage specifies the proportion of the non-MDR TB case population who experience this higher diagnostic rate.

Increased Treatment success (non-MDR)
This increases the treatment success for non-MDR TB cases. The Impact value is implemented as closing the gap between the current value for that year, and 100%. Coverage is implemented as increased case detection.

MDR - increased diagnosis, linkage and treatment success
This increases the rate at which MDR TB cases are diagnosed. The user can specify by what % the diagnostic rate is changed (currently limits are set at -100% to infinity).

The user can also increase the % of diagnosed MDR TB cases that are linked into treatment, implemented as closing the gap between the current value for that year, and 100%.

The % with successful treatment, is also implemented as closing the gap between the current value for that year, and 100%.

The coverage specifies the proportion of the MDR TB case population who experience this higher diagnostic rate, linkage to care or successful treatment.

IPT for HIV positive individuals
We assume IPT provides 35% protection of progression to disease during therapy, assuming that no TST is carried out, following results from the most recent systematic literature review by Akolo et al. and a recent trial on the effect of IPT in a cohort of HIV positive patients receiving ART. (21, 22). This value can be adjusted in the 'Impact' tab. The protection is assumed to stop immediately post therapy. Biologically there is no reason to assume an effect on the risk of progression from a new (re)infection after IPT cessation

therapy. Recent modelling work has suggested that the risk of reactivation is also unaffected in HIV positive individuals, i.e. patients are not cured of their existing infection (23).

IPT coverage is implemented in TIME as part of ACF in HIV+ (option for on and/or off ART). The user can specify the coverage of IPT in each year in the Coverage tab of the Intervention editor. The user will need to specify coverage of ACF in this population, and IPT coverage will fit within the envelope of ACF coverage (e.g. 80% coverage of IPT means 80% of ACF coverage receives IPT). TIME assumes that patients starting on IPT will need to be screened for active TB before the provision of mono-therapy in that time-step and that patients already on IPT from a previous time-step are screened annually for active disease. For simplicity, no adjustment is made to the yield from ACF to account for the lower prevalence in the population already on IPT being rescreened for active disease.

The TB disease probabilities for rapid progression and reactivation for HIV positive individuals are reduced by a factor (1-Imp*Cov). “Imp” is the impact factor (protection from IPT) inputed by the user in the Impact matrix and reflects the impact on progression rates of indivuduals who received IPT. “Cov” is the actual population-level change in the coverage of IPT, compared to the baseline year.

Increased ART coverage

Increases in ART coverage in the general population is modelled through the AIM module in Spectrum. ART coverage from AIM is emulated in Care and Control of TIME Impact so that users can make changes directly in the TIME module. Any changes to ART coverage made in AIM are automatically updated in TIME. TIME Impact assumes that those receiving ART experience the level of protection as specified in the Epidemiology in TIME Impact.

HIV testing and ART initiation

This intervention aims to model the increase in coverage of HIV testing and linkage to ART care for notified TB cases.

In the Implementation/Coverage tab of the Intervention editor, the user can specify the coverage of HIV testing amongst notified TB cases and the proportion of those that are linked into ART care.

The proportion of TB notifications, who are HIV+ and initiated on ART, is calculated by Coverage of test(t)*proportion linked to ART(t) and these are counted as “new ART patients”. Those started on ART experience the treatment success parameters for ART-specific TB cases found in the Care and Control editor as well as the ART parameters specified in the Epidemiology editor.

Active Case Finding

Users can implement active case finding in the general population. Currently active cases finding in high risk groups cannot be modelled because structures for risk groups (other than HIV) are not included in TIME v1.0

ACF is split by HIV category (HIV-, HIV+ not on ART, HIV+ on ART) to allow for targeted ACF in these sub-groups. The user is able to select which HIV stratum should be included in the ACF campaign.

The ACF structure is set up as a copy of the general structure for passive case finding.

The ACF interface requires that the following parameters are entered:

- The sensitivity of the algorithm that is used (for smear positive, smear negative and MDR TB)
- The frequency of ACF campaigns (n per year, duration of 1 round, interval between rounds or the user can specify continuous ACF which is equivalent to 12x 1 month campaigns with no interval between).
- Standard of TB care after diagnosis (relative to the linkage to care and treatment success in the standard care pathway found in the Care and Control tab).

In the coverage tab the user must enter the proportion of the population tested in each round of ACF in each year. For each month in which ACF occurs a proportion of the prevalent pool of TB cases (given by the coverage divided by the length of the round) is tested and those diagnosed (based on specified sensitivity algorithm) are then distributed across the different paths diagnosed TB cases can take (not linked to care, treatment failure, treatment success, etc..).

Preventive Therapy for HIV negative individuals

To reflect successful preventive therapy for HIV negative individuals, a specific post-preventive therapy compartment has been implemented in TIME Impact. In this compartment, individuals are assumed to experience the same risk of reinfection as the rest of the population, but they cannot reactivate, and have the same level of immunity as those in the latent compartment.

The coverage of preventive therapy in HIV- individuals is set to fit within the envelope of the ACF coverage. The user needs to specify the coverage for ACF in HIV- general population in order to be able to model the provision of PT to HIV- individuals.

The user can specify the sensitivity of the chosen test used for screening for LTBI after selecting to include preventive therapy as part of the ACF campaign in the Active case finding tab and the protective efficacy of preventive therapy in HIV- individuals in the Impact tab (default is set 80%, reflecting high adherers in the UATD trial (24)).

In the Coverage tab, the user can specify the proportion of HIV- individuals screened that complete the diagnosis (given that a proportion may be lost to follow-up before LTBI status has been confirmed), the proportion linked to LTBI care and the proportion that complete the full course of preventive therapy.

The proportion of HIV- individuals that move from the latent compartment to the post-preventive therapy compartment is calculated as Coverage ACF(t)*Coverage LTBI Dx(t)*sensitivity*complete DX(t)*Linked to LTBI care(t)*Tx completion rate(t)*protective efficacy. The rest are assumed to remain in the latent compartment.

As current Preventive Therapy include Isoniazid and Rifampicin, the TIME Impact model assumes that MDR latent categories are unaffected by preventive therapy.

IPT for child household contacts of under 5 years old and ACF among household members of all ages

Current WHO policy is to provide all children of under 5 years old who are household contacts of an index case, in the absence of testing for infection. This inherently includes ACF in all household members of all ages.

A systematic review/meta-analysis by Morrison et al (2008) suggests that 30.4% of all household contacts of under 5 years old are infected with Mtb (25). We also assume based on a recent review by Ayieko et al (2014) that the random effects RR of TB disease is 0.55 if completed 6 months of INH compared to those who did not receive IPT (26).

In the intervention editor, the user specifies the coverage of the intervention (ie. the proportion of notified adult cases whose household will be investigated) as well as the proportion of under 5 year old contacts that complete the full course of INH. TIME pulls in the country-specific average number of under 5 year olds per household from the Child Health module.

Since TIME models primary disease as an instantaneous event following infection, the benefit of IPT is split and moves a proportion of u5s from latent and a proportion from susceptible to the post-preventive therapy compartment. This is determined by the proportion of u5 household contacts that are infected with Mtb (30.4%) and the proportion that is either at risk of rapid progression to disease (85%) or at risk of remaining latently infected (15%) (expert opinion).

The number of children under 5 years old that move from the susceptible compartment to the post-preventive therapy compartment is given by (# notified adult cases)*(average # of u5s per household)*(proportion u5 infected)*(proportion progressing rapidly to disease)*(coverage of intervention)*(completion rate)*(protection). This number of u5s are removed from the active disease compartment at the end of the model run in each time-step, before counting the outputs.

The number of children under 5 years old that move from the latent compartment to the post-PT compartment is given by (# notified adult cases)*(average # of u5 per household)*(proportion u5 infected)*(proportion progressing to latent infection)*(coverage of intervention)*(completion rate)*(protection).

Morrison et al. also suggest in their systematic review/meta-analysis that 4.5% of household contacts of an index case have active disease (25). For the ACF component, the user will need to specify the coverage of the intervention (ie. the proportion of notified adult cases whose household will be investigated, which will be the same as specified above), details for the screening algorithm (sensitivity, relative detection of SS- and relative detection of MDR) as well as the relative linkage to care and treatment success.

The average household size is pulled into TIME from the Child Health module. Additional cases picked up in the ACF is given by (# of notified adult cases)*(average household size-1)*(coverage of intervention)*(proportion active disease)*(sensitivity of algorithm) and these will enter the ACF care pathway.

The ACF component is applied before the IPT in u5s in order to reflect the removal of active cases in that age group before the provision of IPT and to avoid the provision of mono-therapy to active cases.

8. Summary health measures

TIME v1.0 calculates summary health measures which are presented as absolute number of years of life lived with disability (YLD), years of life lost (YLL) and disability-adjusted life years (DALY).

TIME bases its calculations on the following formulae:

$$YLD = \sum_t \sum_i \sum_j (N_{tij}) \times w_i \times (1+d)^{1-t}$$

$$YLL = \sum_t \sum_i \sum_j (D_{tij}) \times LE_j \times (1+d)^{1-t}$$

Where, $DALY = YLD + YLL$ and therefore $DALYs\ averted\ (\Delta DALY) = \Delta YLD + \Delta YLL$

t = year (where first year count starts at 1).
i = health state stratified by TB (active vs. no disease), HIV status (HIV+ vs. HIV-), CD4 category (<50, 50-99, 100-199, 200-249, 250-349, 350-499, >500) and length of time on ART (no ART, 0-6 months, 7-12 months, >12 months).
j = age group.
N_{tij} = The number of individuals (prevalence) in a health state in a given year and age group.
D_{tij} = Number of all-cause deaths in each health state in a given year and age group.
LE_j = The life expectancy for the midpoint in a given age group. Default is to use country-specific life tables stored in DemProj, but users can select the option for using a standardized life table adapted from Murray et al 2010.(27)
d = discount rate. Default is set to 0, but can be adjusted by putting in the discount rate in the form of a proportion in the configuration window (e.g. a discount rate of 3% should be inputted as 0.03).
w_i = disability weight for each health state (Table 4) adapted from Salomon et al. 2012.(28)

Table 4. Disability weights by health state

<table>
<tr><th colspan="3">HIV</th><th colspan="2">TB</th></tr>
<tr><th colspan="3">HIV state</th><th>Active TB</th><th>Non active TB</th></tr>
<tr><td colspan="3">HIV negative</td><td>0.331</td><td>0.000</td></tr>
<tr><td rowspan="7">HIV positive not on ART</td><td rowspan="7">CD4 sub-category (cells/uL)</td><td>CD4 < 50</td><td rowspan="3">0.547</td><td rowspan="3">0.547</td></tr>
<tr><td>CD4 50-99</td></tr>
<tr><td>CD4100-199</td></tr>
<tr><td>CD4 200-249</td><td rowspan="2">0.399</td><td rowspan="2">0.221</td></tr>
<tr><td>CD4 250-349</td></tr>
<tr><td>CD4 350-499</td><td rowspan="2">0.331</td><td rowspan="2">0.054</td></tr>
<tr><td>CD4 > 500</td></tr>
<tr><td colspan="2" rowspan="3">HIV positive on ART</td><td>0-6 months</td><td rowspan="3">0.331</td><td rowspan="3">0.053</td></tr>
<tr><td>7-12 months</td></tr>
<tr><td>> 12 months</td></tr>
</table>

Data for CD4-specific disability weights is available for three CD4 categories (Table 4); therefore, we assume the same disability weight across CD4 categories as stratified in TIME, based on available data (i.e. there is no evidence to support interpolation between categories). Furthermore, we assume a constant disability weight regardless of length of time on ART, as per available data.

TIME v1.0 provides absolute number of years of life lived with disability, years of life lost and DALYs, by year and stratified by age group. The user will need to make use of external software (such as Excel) for further analysis of the outputs, such as calculation of DALYs averted due to an intervention, or the distribution of DALYs between children vs. adults.

9. Demography

TIME Impact uses parameters from DemProj in order to create the modelled population. Births are introduced into the model and calculated directly in TIME by applying the ASFR and TFR from DemProj to women of 15-49 years old. Ageing rates are calculated by taking the population of the final age (eg. Population of 9 year olds) in each age-bin and dividing it by the population of all ages in the age-bin (eg. Population of 5-9 year olds). Background mortality is calculated using life tables. The number of deaths in each age-bin is divided by the mid-year population of that age-bin to calculate the mortality rate. TB deaths

from TIME Impact are removed from overall background mortality in order to avoid double counting. International migration is currently ignored in TIME's demographic model.

10. Model Initialisation and population size adjustments

Model initialisation

The model is initialised in 3 phases which aim to create a stable TB epidemic in 1970 (pre-HIV) with approximately correct demographic composition and dynamics (birth and death rates).

Phase 1: Demographics

The 1970 age and sex distribution, together with TFR and ASFR as well as age specific death rates derived from life tables is used and run for 400 years to create an equilibrium/stable age structured population for 1970.

Phase 2: TB

A 100 active TB cases are introduced into the population, with TB epidemiology parameters as set and the care and control parameter values as set for 1970. The model is then run for another 400 years to achieve equilibrium/stable disease state.

Phase 3: Adjusting to fit 1970 population

The population, which will be stable in age structure and TB incidence, is then adjusted to match the age and sex structure in 1970. In this adjustment, the age distribution of TB cases is maintained as in stage 2. Adjustment can be turned off in the configuration window starting at year 1970.

The final population is then started in 1970 with the UN birth and death rates as well as the TB care and control parameters values in 1970.

Phase 4: MDR initialisation

The parameters are set to 0 until MDR is introduced into the population. The user can specify the date of MDR introduction, starting at 1971, and the proportion of retreatment and new cases that is MDR at that time.

11. Fitting

Fitting

The fitting of TIME to a country's specific TB epidemic profile is currently being done manually during the development phase to improve understanding of model behaviour. There is currently no automatic fitting algorithm that takes a model with default values and approximates a fully fitted model.

12. References

1. Stover J, Andreev K, Slaymaker E, Gopalappa C, Sabin K, Velasquez C, et al. Updates to the spectrum model to estimate key HIV indicators for adults and children. AIDS. 2014;28 Suppl 4:S427-34.
2. Stover J, Brown T, Marston M. Updates to the Spectrum/Estimation and Projection Package (EPP) model to estimate HIV trends for adults and children. Sex Transm Infect. 2012;88(Suppl 2):i11-i6.
3. Stover J, Johnson P, Hallett T, Marston M, Becquet R, Timaeus IM. The Spectrum projection package: improvements in estimating incidence by age and sex, mother-to-child transmission, HIV progression in children and double orphans. Sex Transm Infect. 2010;86(Suppl 2):ii16-ii21.
4. Espinal MA, Kim SJ, Suarez PG, Kam KM, Khomenko AG, Migliori GB, et al. Standard short-course chemotherapy for drug-resistant tuberculosis: treatment outcomes in 6 countries. JAMA. 2000;283(19):2537-45.
5. Organization WH. Global Tuberculosis Report 2014. Geneva: WHO, 2014 WHO/HTM/TB/2014.08.
6. Menzies NA, Cohen T, Lin HH, Murray M, Salomon JA. Population health impact and cost-effectiveness of tuberculosis diagnosis with Xpert MTB/RIF: a dynamic simulation and economic evaluation. PLoS Med. 2012;9(11):e1001347.
7. Dowdy DW, Chaisson RE. The persistence of tuberculosis in the age of DOTS: reassessing the effect of case detection. Bull World Health Organ. 2009;87(4):296-304.
8. Dye C, Garnett GP, Sleeman K, Williams BG. Prospects for worldwide tuberculosis control under the WHO DOTS strategy. Directly observed short-course therapy. Lancet. 1998;352(9144):1886-91.
9. Sonnenberg P, Glynn JR, Fielding K, Murray J, Godfrey-Faussett P, Shearer S. How soon after infection with HIV does the risk of tuberculosis start to increase? A retrospective cohort study in South African gold miners. J Infect Dis. 2005;191(2):150-8.
10. Williams BG, Granich R, De Cock KM, Glaziou P, Sharma A, Dye C. Antiretroviral therapy for tuberculosis control in nine African countries. Proc Natl Acad Sci U S A. 2010;107(45):19485-9.
11. Murray CJ, Salomon JA. Modeling the impact of global tuberculosis control strategies. Proc Natl Acad Sci U S A. 1998;95(23):13881-6.
12. Behr MA, Warren SA, Salamon H, Hopewell PC, Ponce de Leon A, Daley CL, et al. Transmission of Mycobacterium tuberculosis from patients smear-negative for acid-fast bacilli. Lancet. 1999;353(9151):444-9.
13. Getahun H, Harrington M, O'Brien R, Nunn P. Diagnosis of smear-negative pulmonary tuberculosis in people with HIV infection or AIDS in resource-constrained settings: informing urgent policy changes. Lancet. 2007;369(9578):2042-9.
14. Lew W, Pai M, Oxlade O, Martin D, Menzies D. Initial drug resistance and tuberculosis treatment outcomes: systematic review and meta-analysis. Ann Intern Med. 2008;149(2):123-34.
15. Lawn SD, Kranzer K, Wood R. Antiretroviral therapy for control of the HIV-associated tuberculosis epidemic in resource-limited settings. Clin Chest Med. 2009;30(4):685-99, viii.
16. Badri M, Wilson D, Wood R. Effect of highly active antiretroviral therapy on incidence of tuberculosis in South Africa: a cohort study. Lancet. 2002;359(9323):2059-64.
17. Eamranond P, Jaramillo E. Tuberculosis in children: reassessing the need for improved diagnosis in global control strategies. The International Journal of Tuberculosis and Lung Disease. 2001;5(7):594-603.
18. Styblo K. Epidemiology of tuberculosis in children. Bull Int Union Tuberc Lung Dis. 1982;57:133-9.
19. Praygod G, Todd J, McDermid J. Early childhood tuberculosis in northwestern Tanzania. The International Journal of Tuberculosis and Lung Disease. 2012;16(11):1455-60.
20. Dodd PJ, Gardiner E, Coghlan R, Seddon JA. Burden of childhood tuberculosis in 22 high-burden countries: a mathematical modelling study. The lancet global health. 2014;2(8):e453-e9.
21. Akolo C, Adetifa I, Shepperd S, Volmink J. Treatment of latent tuberculosis infection in HIV infected persons. Cochrane Database Syst Rev. 2010(1):CD000171.
22. Rangaka MX, Wilkinson RJ, Boulle A, Glynn JR, Fielding K, van Cutsem G, et al. Isoniazid plus antiretroviral therapy to prevent tuberculosis: a randomised double-blind, placebo-controlled trial. Lancet. 2014;384(9944):682-90.

23. Houben RM, Sumner T, Grant AD, White RG. Ability of preventive therapy to cure latent Mycobacterium tuberculosis infection in HIV-infected individuals in high-burden settings. Proceedings of the National Academy of Sciences of the United States of America. 2014;111(14):5325-30.
24. Smeija MJ, Marchetti CA, Cook DJ, F.M. S. Isoniazid is effective in helping to prevent tuberculosis in people not infected with HIV. Cochrane Database Syst Rev. 1999.
25. Morrison J, Pai M, Hopewell PC. Tuberculosis and latent tuberculosis infection in close contacts of people with pulmonary tuberculosis in low-income and middle-income countries: a systematic review and meta-analysis. The Lancet infectious diseases. 2008;8(6):359-68.
26. Ayieko J, Abuogi L, Simchowitz B, Bukusi EA, Smith AH, Reingold A. Efficacy of isoniazid prophylactic therapy in prevention of tuberculosis in children: a meta-analysis. BMC Infect Dis. 2014;14(1):91.
27. Murray CJ, Ezzati M, Flaxman AD, Lim S, Lozano R, Michaud C, et al. Supplementary appendix to: Comprehensive systematic analysis of global epidemiology: Definitions, methods, simplification of DALYs, and comparative results from the Global Burden of Disease Study 2010. Lancet. 2012;380.
28. Salomon JA, Vos T, Hogan DR, Gagnon M, Naghavi M, Mokdad A, et al. Common values in assessing health outcomes from disease and injury: disability weights measurement study for the Global Burden of Disease Study 2010. The Lancet. 2013;380(9859):2129-43.
